# Glowing Seashells: Diversity of Fossilized Coloration Patterns on Coral Reef-Associated Cone Snail (Gastropoda: Conidae) Shells from the Neogene of the Dominican Republic

**DOI:** 10.1371/journal.pone.0120924

**Published:** 2015-04-01

**Authors:** Jonathan R. Hendricks

**Affiliations:** Department of Geology, San José State University, California, United States of America and Paleontological Research Institution, Ithaca, New York, United States of America; University of California, UNITED STATES

## Abstract

The biology of modern Conidae (cone snails)—which includes the hyperdiverse genus *Conus*—has been intensively studied, but the fossil record of the clade remains poorly understood, particularly within an evolutionary framework. Here, ultraviolet light is used to reveal and characterize the original shell coloration patterns of 28 species of cone snails from three Neogene coral reef-associated deposits from the Cibao Valley, northern Dominican Republic. These fossils come from the upper Miocene Cercado Fm. and lower Pliocene Gurabo Fm., and range in age from about 6.6-4.8 Ma. Comparison of the revealed coloration patterns with those of extant species allow the taxa to be assigned to three genera of cone snails (*Profundiconus*, *Conasprella*, and *Conus*) and at least nine subgenera. Thirteen members of these phylogenetically diverse reef faunas are described as new species. These include: *Profundiconus*? *hennigi*, *Conasprella (Ximeniconus) ageri*, *Conus anningae*, *Conus lyelli*, *Conus (Atlanticonus*?) *franklinae*, *Conus (Stephanoconus) gouldi*, *Conus (Stephanoconus) bellacoensis*, *Conus (Ductoconus) cashi*, *Conus (Dauciconus) garrisoni*, *Conus (Dauciconus*?) *zambaensis*, *Conus (Spuriconus*?) *kaesleri*, *Conus (Spuriconus*?) *lombardii*, *and Conus (Lautoconus*?) *carlottae*. Each of the three reef deposits contain a minimum of 14–16 cone snail species, levels of diversity that are similar to modern Indo-Pacific reef systems. Finally, most of the 28 species can be assigned to modern clades and thus have important implications for understanding the biogeographic and temporal histories of these clades in tropical America.

## Introduction

**Fig 1:**
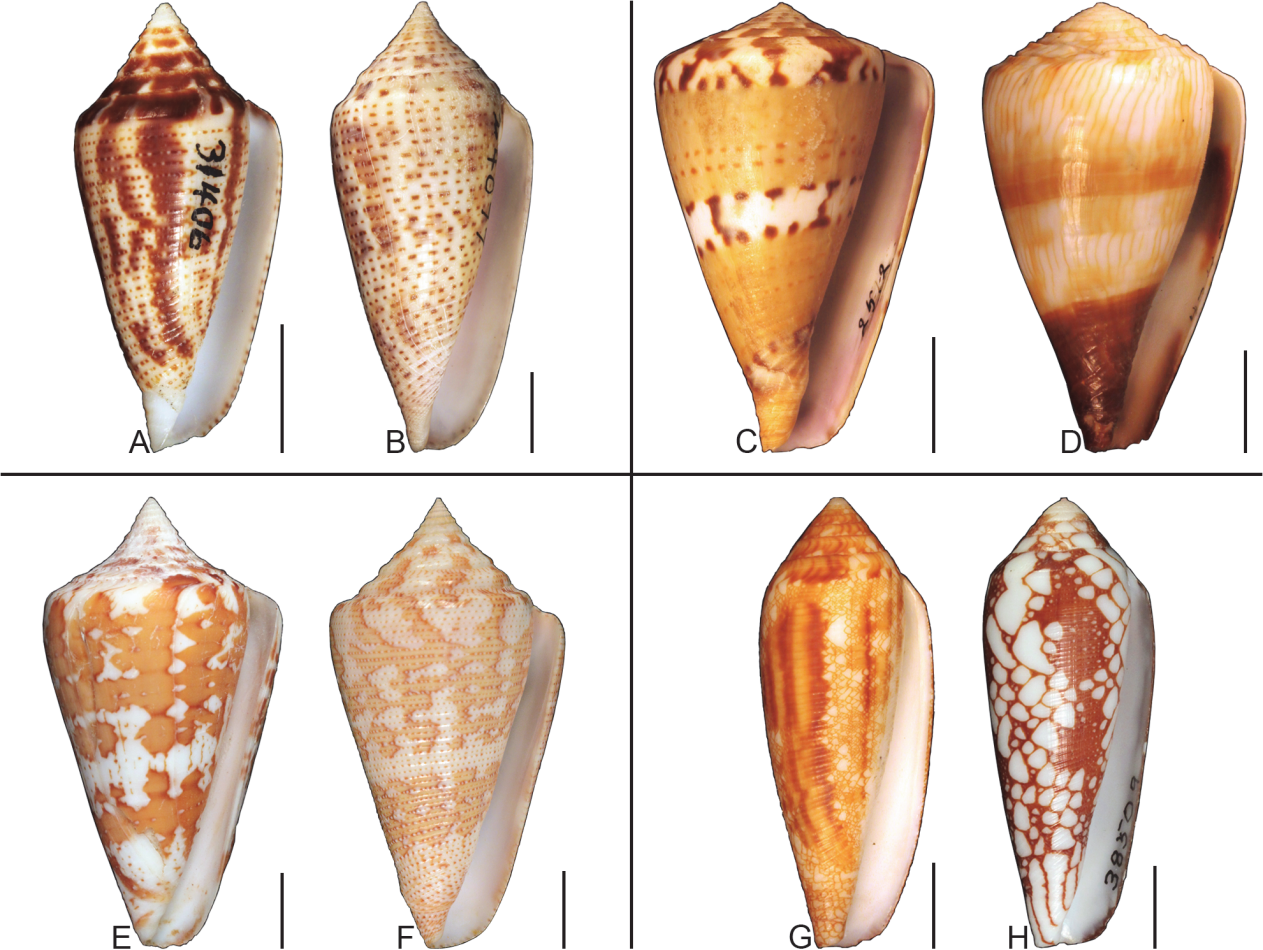
Variation in shell coloration patterns between pairs of closely related cone snail species. Members of selected pairs belong to the same subgenus (based on phylogeny and classification of Puillandre et al. [1, 2]). (A) *Conasprella* (*Ximeniconus*) *mahogani* (CASIZ 184267); (B) *Conasprella* (*Ximeniconus*) *ximenes* (CASIZ 184268); (C) *Conus* (*Rhizoconus*) *capitaneus* (CASIZ 178062); (D) *Conus* (*Rhizoconus*) *miles* (CASIZ 178070); (E) *Conus* (*Stephanoconus*) *archon* (UF 26040); (F) *Conus* (*Stephanoconus*) *mappa* (UF 238779); (G) *Conus* (*Darioconus*) *auricomus* (CASIZ 178034); (H) *Conus* (*Darioconus*) *episcopatus* (CASIZ 180453). All scale bars are 1 cm.

Cone snails—which include three small genera, *Profundiconus*, *Californiconus*, and *Conasprella*, and one large genus, *Conus*—are one of the most well-studied and species-rich clades of marine animals, and are represented by over 700 extant species [1, 2]. The diverse and often intricate shell coloration patterns of cone snails (e.g., Fig. 1), which all belong to the venomous neogastropod family Conidae [3], have caused them to be coveted by shell collectors for centuries, and these patterns have even been the subjects of studies by computer modelers (e.g., [4, 5]). Besides being appealing to look at, the coloration patterns of cone snail shells are taxonomically useful for distinguishing extant species. While species exhibit intraspecific variation in the finer details of their coloration patterns (e.g., [6–8]), the major elements that make up a given pattern—for example, presence of spiral rows of dots (Fig. 1A,B) or unpigmented tents (Fig. 1G,H)—do not tend to vary substantially within species. Furthermore, cone snail shell coloration patterns seem to contain phylogenetic signal because closely related species often show subtle variations on the same general themes of patterning (Fig. 1).

**Fig 2:**
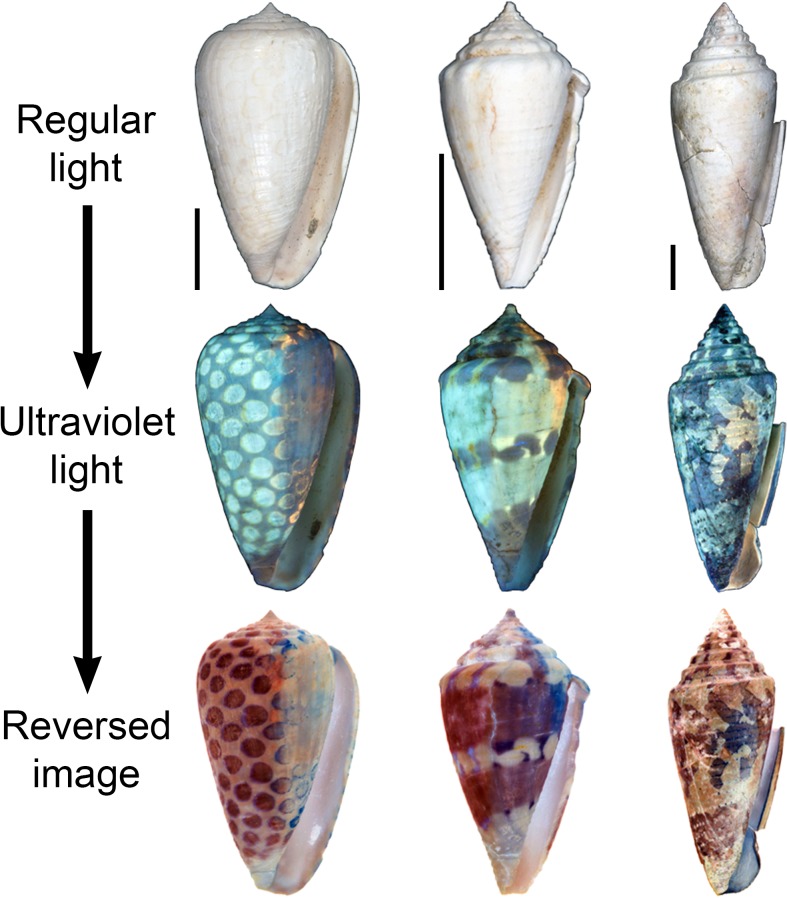
Examples of ancient coloration patterns revealed by UV light. Top row: three fossil shells (PRI 66199, PRI 66166, and PRI 66158) from the Neogene of the Dominican Republic photographed under regular light; all scale bars are 1 cm. Middle row: the same specimens photographed under longwave UV light. Bottom row: reversed images that show how fluorescing regions correspond to parts of the shell that were once darkly pigmented.

In addition to being remarkably diverse today, cone snails have a substantial fossil record that extends back to the early Eocene [9]. Over 1,000 species have been described from the fossil record [6], though many of these are likely synonymous (e.g., [10, 11]). This is at least in part because cone snail species are relatively conservative in shell morphology and fossils seemingly lack the rich suite of coloration patterns that are often helpful for distinguishing extant species. In the 1960’s, however, Axel Olsson [12] discovered that original coloration patterns—if not the actual colors themselves—sometimes become visible when fossil shells are illuminated with ultraviolet (UV) light, which causes formerly pigmented regions of the shell to fluoresce (also see [13]). Many authors have employed this approach as an aid for characterizing Cenozoic mollusk fossils (e.g., [14–17]), including cone snails (e.g., [11–13, 18–21]). Once a specimen has been photographed under UV light, it is possible to use photoediting software to reconstruct the original shell coloration pattern. This is done by creating a reversed, negative image, which causes bright, fluorescing markings to become darkened, corresponding with regions that were once pigmented (e.g., Fig. 2; also see [13]).

While the use of UV light to reveal ancient shell coloration patterns has proved to be a useful technique for understanding the systematics of some fossil mollusks, we still do not have a clear understanding of exactly what compounds are responsible for pigmentation in modern shells [22, 23], much less what matter is actually fluorescing in the fossil shells. Hedegaard et al. [23] recently used resonance Raman microspectrometry to study the shell pigments of a variety of mollusks—including the cone snail *Conus marmoreus*—and demonstrated that “[t]he colour of a given shell is possibly due to several compounds, at least one of which is often a polyene” (p. 161). These authors cautioned, however, that “there is no trivial relationship between colour, pigment and taxon” (p. 157). Clearly much more work needs to be done to better understand the compounds responsible for shell coloration.

**Fig 3:**
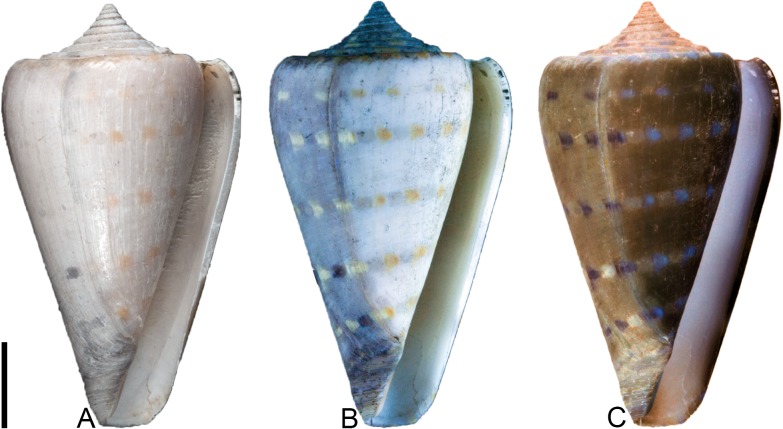
Correspondence between oxidation of the shell, fluorescence under UV light, and effects of digitally reversing the image of the fluorescing shell using photoediting software. Shell is a specimen of *Conus humerosus* (PRI 67550); scale bar is 1 cm. See text for explanation.

That said, it seems that oxidation—caused either naturally by exposure of shells to sunlight over prolonged periods of time, or artificially by soaking in bleach for several days—plays a role in causing formerly pigmented regions of fossil shells to fluoresce under UV light [13], though the reason is not currently understood. A fossil specimen of *Conus humerosus* Pilsbry, 1921 (Fig. 3) provides a useful demonstration of the relationship between pigmented parts of the shell and fluorescence induced by UV light. Under regular light (Fig. 3A), the right side of the ventral surface of the last whorl shows five spiral rows of orange-colored, square-shaped spots; these are not visible on the left side. Under UV light (Fig. 3B), however, the same spiral rows of spots fluoresce, becoming visible on the left side (Fig. 3C shows a reversed version of Fig. 3B). This implies that prior to collection, the right side of the specimen remained buried in the sediment, while the left side was exposed to the light of the sun and consequently oxidized. It should be noted that most fossil cone snail shells show little or no evidence of their original coloration patterns under regular light; *Conus humerosus* is one of the few known species that typically does, suggesting that its shell was heavily pigmented in life.

UV light is used here to reveal and characterize the great diversity of shell coloration patterns exhibited by fossil cone snails from three Neogene-aged, coral reef-associated assemblages from the Cibao Valley of the northern Dominican Republic (DR), the geology and paleontology of which has been intensively studied (e.g., [24–26]). These three assemblages were selected for their diversity of species and quality of preserved coloration patterns, which permit 13 new species to be recognized and described. The coloration patterns of 15 previously described species are also characterized here, but comprehensive taxonomic treatments (including complete re-descriptions) of these will be published in a future work. Finally, the preserved coloration patterns documented here allow many of the newly and previously studied species to be assigned to modern clades, providing insight on the phylogenetic and biogeographic history of cone snails in tropical America.

## Materials and Methods

### Collection Localities

The study specimens were collected in the 1970’s by Harold and Emily Vokes of Tulane University (TU) and are from three TU collecting stations—TU 1422, TU 1354, and TU 1215—that were characterized by E. Vokes [27] as "true [coral] reef localities" (p. 16). This is true for TU 1422 and TU 1354, but see remarks below for TU 1215, which may represent a mixed assemblage.

The first locality, TU 1422, is located at Arroyo Bellaco, “which is a tributary of Río Cana from the east, [a] coral reef that is exposed for approximately 1 km below the ford at Las Caobas Adentro, 3 km southwest of Las Caobas” ([24], p. 67). Klaus and Budd [28] intensively studied the coral reef at this locality, which was described by Vokes [27] as “the most spectacular reef of all” (p. 16). Based on the map of Klaus and Budd ([28], Fig. 1), the east end of the locality is at ca. 19.483065°, -71.246228° and the west end at 19.485386°, -71.250524° (this locality was georeferenced using Google Earth, as were TU 1354 and TU 1215). TU 1422 is positioned in the upper Miocene Cercado Fm. and thus formed sometime between 6.0–6.6 Ma [26]. Klaus and Budd [28] reported 35 coral species from this locality, five of which are extant.

Locality 1354 is located along “Cañada de Zamba, a tributary on the west side of the Río Cana, approximately 2.5 km east of the village of Zamba, which is 7 km north of Cruz de Santiago (Santiago Rodriguez), on road to Guayubin; or 4.5 km (airline) below the ford at Caimito” ([24], p. 66). The boundaries of the locality are shown in Saunders et al. ([24], text-Fig. 15); the east end of the locality is at ca. 19.542518°, -71.300043° and the west end at 19.540455°, -71.303286°. According to Waller [29], TU 1354 is positioned in the lower Pliocene Gurabo Fm. at about 335–345 m in the section of Saunders et al. [24]. Based on the age-depth model of McNeill et al. [26], this locality is ca. 4.9 Ma. Nearly 60 coral species have been reported from NMB localities equivalent to TU 1354 (based on data available at http://nmita.geology.uiowa.edu). Waller ([29], p. 124) characterized the paleoenvironment of TU 1354 as “apparently a shallow but clear-water phase of the Gurabo with calcareous silts and corals.”

Finally, locality TU 1215 is exposed at bluffs on both sides of Río Gurabo, “from the ford on Los Quemados-Sabaneta road, upstream to approximately 1 km above the ford” ([24], p. 64). The boundaries of the locality are shown in Sanders et al. ([24], text-Fig. 5); the north end of the locality is at ca. 19.506678°, -71.182946°, and the south end at 19.498825°, -71.179809°. According to Waller [29], TU 1215 is positioned in the lower Pliocene Gurabo Fm., between about 275–384 m in the section of Saunders et al. [24]. This is a large collecting locality and—according to the Río Gurabo age-depth model of McNeill et al. [26]—ranges from ca. 4.8–5.4 Ma. Over 60 coral species have been reported from NMB localities equivalent to TU 1215 (based on data available at http://nmita.geology.uiowa.edu). There is debate about whether the corals are preserved *in situ*, as suggested by Vokes [27], or if they “tumbled downslope into fine sediments” (Waller [29], p. 29). Based on the fauna of pectinids at TU 1215, Waller [29] argued that the locality likely represents water depths of around 50 m or more. Given this, it is possible that the cone snail fauna at this locality represents more than one paleoenvironmental setting (deeper water species with transported reef-associated species) and—given the ca. 0.6 My duration of the locality—potentially taxa that did not co-exist in time.

### Museum Collections

All of the 359 specimens reported on here from TU stations 1215, 1354, and 1422 are reposted at the Paleontological Research Institution (PRI) in Ithaca, New York and listed in S1 Table. Additional specimens—figured and/or referred to—are from the: Invertebrate Paleontology Collection at the Academy of Natural Sciences of Drexel University (ANSP) in Philadelphia, Pennsylvania; Department of Invertebrate Zoology and Geology, California Academy of Sciences (CASIZ) in San Francisco; Division of Invertebrate Zoology, Florida Museum of Natural History at the University of Florida (UF) in Gainesville; Fossil Invertebrate Collections, Natural History Museum, London (NHMUK PI); and Geosciences Collection, Naturhistorisches Museum Basel (NMB), Switzerland. As other workers collected all of the fossil specimens considered here several decades ago, no permits were required for the described study, which complied with all relevant regulations.

### Specimen Preparation

Because the specimens come from unconsolidated deposits, removal from rock matrix was not necessary. All but the most fragile specimens were gently scrubbed under water to remove attached sand and silt particles and were then soaked overnight in a solution of diluted (50%) Clorox bleach. The shells were then rinsed again in water and allowed to dry. Next, the shells were inspected for preserved coloration patterns using a Raytech LS-7CB UV lamp in a dark room. Most of the shells studied show no indication of their coloration patterns under regular light, but approximately 60% showed well-preserved evidence of their ancient coloration patterns when viewed under UV light. A majority of the specimens exhibiting fluorescing coloration patterns were selected for digital photography.

### Digital Photography and Image Processing

Most specimens were photographed using either a Canon 50D or Nikon 7100D digital camera attached to a copy stand. Black sand was used as a mounting medium in order to ensure that specimens were photographed in standardized positions (most often as views of the ventral or dorsal surface, with the axis of coiling oriented perpendicular to the optical axis of the camera lens; in apical views, the axis of coiling is parallel to the optical axis of the lens). Two Raytech LS-7CB lamps were used for UV photography; they were set to emit longwave UV light (365 nm) and were positioned on either side of the specimen being photographed. In order to block out background light during UV photography, photographs were either taken in a dark room, or a large box was used to cover the entire photography apparatus. Figures were prepared using Adobe Photoshop CS6. Adjustments were made to the white balance and levels of individual images, and some images were also treated using the auto tone, auto contrast, and/or auto color functions of Photoshop. Images of most specimens photographed under UV light were digitally reversed (using Photoshop’s invert function) to reconstruct shell coloration patterns (see above). Importantly, these reversed images do not show the actual shell colors of the animal when it was alive. Showing these reversed images in color (as opposed to grayscale) is useful, however, because differences in color—especially between different elements of pigmentation—on the same shell may correspond with variability in the underlying organic molecules that are fluorescing under UV light and/or the taphonomic (i.e., postmortem) history of the individual specimen. Unaltered, original digital images of specimens photographed under UV light and figured herein are freely available for inspection and download at DataDryad.org (doi:10.5061/dryad.n36n6). These include uncompressed raw .CR2 (Canon) and .NEF (Nikon) files, as well as corresponding .JPEG files; each of the raw files retains information about the camera settings (e.g., F numbers and exposure times) used to produce the photograph.

### Recognition, Characterization, and Treatment of Species

The taxonomic monographs of Röckel et al. [6] and Kohn [8] have quantitatively and qualitatively demonstrated the ranges of variability that extant cone snail species exhibit in the different features of their shells and thus serve as useful benchmarks for differentiating intra- from interspecific variation in fossil species, which is always a challenge in paleontology [30]. Although morphological features of the shell may not allow recognition of cryptic species of cone snails [31, 32], all attempts were made here to circumscribe species in a manner consistent with the treatments of Röckel et al. [6] and Kohn [8], which accepted substantial amounts of variability in certain aspects of shell morphology (e.g., shape and minor details of coloration patterning). These authors defined species broadly, resulting in many previously described forms—many based on a very small number of specimens—being synonymized. This work follows such a non-typological approach to the circumscription of cone snail species (also see [11]).

Prior to beginning this project, all available type specimens of previously described species from the Neogene of the Dominican Republic were observed in person and photographed. The new species described herein were compared with these to ensure that they had not previously been described. Species were only formally described as new if at least three specimens were available. This is an admittedly arbitrary cutoff, but ensures at least a minimal characterization of intraspecific variability (including ontogenetic changes in form, which may be taxonomically important and cannot usually be characterized with only two specimens); a higher threshold would prevent description of some species that were naturally rare. Some distinctive morphologies were observed that do not meet this threshold; while some of these likely represent additional new species, they are not formally described at this time (some are figured here, however). Importantly, many of these are small specimens and may thus represent juveniles of species that have already been described, an issue recently also encountered by Kohn [8].

### Nomenclatural Acts

The electronic edition of this article conforms to the requirements of the amended International Code of Zoological Nomenclature, and hence the new names contained herein are available under that Code from the electronic edition of this article. This published work and the nomenclatural acts it contains have been registered in ZooBank, the online registration system for the ICZN. The ZooBank LSIDs (Life Science Identifiers) can be resolved and the associated information viewed through any standard web browser by appending the LSID to the prefix "http://zoobank.org/". The LSID for this publication is: urn:lsid:zoobank.org:pub:1B73A54D-BF92-4BB2-98DE-F3FC9D80E753. The electronic edition of this work was published in a journal with an ISSN, and has been archived and is available from the following digital repositories: PubMed Central and LOCKSS.

### Morphological Terminology

Morphological terminology generally follows Röckel et al. [6], Hendricks [11], and Kohn [8]. An exception is the treatment of coloration patterning. Cone snail shell coloration patterns often show multiple pigmentation elements (e.g., dots, blotches, or bands) that *appear* to be positioned in different layers—that is, in superimposition—relative to each other. For example, a shell may have a base of pigmented blotches that appears to be covered by numerous spiral rows of dots (e.g., Fig. 1B). Another example is a shell that has a base of axial streaks that appears to be overprinted by solid spiral bands (e.g., Fig. 1D). This study found that treating pigmentation elements as though they occur in separate (but sometimes interacting) layers was useful for their characterization and description. While covered layers are described here as “basal” or “primary” relative to the “secondary” layers that cover them, this is not meant to imply anything about the actual ordering of pigment deposition into the shell. Furthermore, it is important to note that experimental work on modern cone snail shells will be required to determine whether different pattern elements on the same shell actually exist in separate layers relative to each other, as is the case in at least one species of *Littorina* [33]. The terminology for individual pigmentation elements follows that of Röckel et al. [6]. In some cases, shell coloration patterns revealed by UV fluorescence exhibit more than one color of emitted light. Such cases are noted here, as these variations may correspond with differences in the pigments that once colored the unfossilized shell; confirmation of this, however, will also require experimental work.

Abbreviations used in reference to shell measurements are from Röckel et al. [6] and include: SL, shell length; MD, maximum diameter; HMD, height of maximum diameter; and AH, aperture height (also see [8, 11]). From these, the following ratios were calculated to characterize different aspects of shell shape: RD, relative diameter (= MD/AH); PMD, position of maximum diameter (= HMD/AH); and RSH, relative spire height (= [SL-AH]/SL). See Röckel et al. [6] and Kohn [8] for qualitative descriptions of these metrics, which are also used here. Qualitative descriptions of shell size—based on maximum observed SL—follow Röckel et al. [6]: small, 15–25 mm; moderately small, 25–35 mm; medium, 35–55 mm; moderately large, 55–80 mm; and large, >80 mm. Measurement values for specimens belonging to newly described species are given in S2 Table.

### Classification

This study follows the recent cone snail classification published by Puillandre et al. [2], which is based upon molecular phylogenetic evidence [1]. This classification divides extant cone snails among four genera (*Profundiconus*, *Californiconus*, *Conasprella*, and *Conus*) and numerous subgenera (most of which were recognized as genera by Tucker and Tenorio [34]). While taxonomic classifications above the species level are largely arbitrary (e.g., [2, 35, 36]), the decision by Puillandre et al. [2] to split cone snails into four genera is based on their identification of several deep, ancient phylogenetic divergences that divide cone snails into four main clades [1]. Although debate may continue about whether a given cone snail clade should be recognized at the Linnaean rank of subgenus, genus, or family, such discussions are largely irrelevant to study of evolution in a phylogenetic context [35], unless one presumes (or requires) that equally-ranked taxa should be directly comparable in some way that is biologically meaningful. The rankings employed are very important, however, as tools for communication and in this respect the four-genus system of Puillandre et al. [1, 2] is appealing from the perspective of nomenclatural stability, as cone snails have long been treated by many workers as belonging to one genus, *Conus* (see overview in [6]), and most extant species retain this generic assignment in the new classification [2].

The fossil species considered here are not placed in an explicit phylogenetic context at this time. Nevertheless, combinations of shell features shared between extant species of known phylogenetic position and the fossil species allow predictions to be made about the relationships of many of the extinct species to members of the modern fauna. Where possible, the fossil species have been assigned to both a genus and subgenus of cone snails. When this has not been possible, species have been assigned to the genus *Conus*. Unidentified fossils are referred to as Conidae sp., though most of these likely belong to *Conus*.

## Systematics

In total, the cone snail fauna comprising TU stations 1422, 1215, and 1354 includes at least 28 species (Table 1), 13 of which are newly described below. Information about the type species for each higher taxon comes from Puillandre et al. [2] or Tucker and Tenorio [34]. See Tucker and Tenorio [34] for information about the fossil record and morphological characteristics of these higher taxa. Abbreviated synonymy lists are provided for previously described species.

**Table 1:** Occurrence data.

Species	TU 1422	TU 1215	TU 1354	Total
*Conasprella* (*Dalliconus*) cf. *sauros* (Garcia, 2006)		15		15
*Conasprella* (*Ximeniconus*) *ageri* sp. nov.	2		28	30
*Conasprella* (*Ximeniconus*) *cercadensis* (Maury, 1917)	4	1		5
*Conasprella* (*Ximeniconus*) *kitteredgei* (Maury, 1917)			4	4
*Conasprella stenostoma* (Sowerby I, 1850)		2		2
*Conus* ("*Pyruconus*") *haytensis* Sowerby I, 1850		6		6
*Conus* (*Atlanticonus*?) *franklinae* sp. nov.	3	1		4
*Conus* (*Atlanticonus*?) *olssoni* Maury, 1917	10	14	10	34
*Conus* (*Dauciconus*?) *zambaensis* sp. nov.			15	15
*Conus* (*Dauciconus*) *furvoides* Gabb, 1873			5	5
*Conus* (*Dauciconus*) *garrisoni* sp. nov.	4			4
*Conus* (*Dauciconus*) *karlschmidti*? Maury, 1917		9		9
*Conus* (*Dauciconus*) *multiliratus* Böse, 1906		7	1	8
*Conus* (*Dauciconus*) *planiliratus* Sowerby I, 1850		3	10	13
*Conus* (*Ductoconus*) *cashi* sp. nov.	2	1		3
*Conus* (*Lautoconus*?) *carlottae* sp. nov.	2		13	15
*Conus* (*Pyruconus*) *recognitus* Guppy, 1866			6	6
*Conus* (*Spuriconus*?) *kaesleri* sp. nov.			7	7
*Conus* (*Spuriconus*?) *lombardii* sp. nov.	20			20
*Conus* (*Spuriconus*) *humerosus* Pilsbry, 1921	1	2	1	4
*Conus* (*Spuriconus*) *spurius* Gmelin, 1791			4	4
*Conus* (*Stephanoconus*) *bellacoensis* sp. nov.	2		1	3
*Conus* (*Stephanoconus*) *gouldi* sp. nov.	6			6
*Conus* (*Stephanoconus*) *sewalli* Maury, 1917	5	3	1	9
*Conus anningae* sp. nov.		3		3
*Conus lyelli* sp. nov.	5		2	7
*Conus symmetricus* Sowerby I, 1850	8	43	30	81
*Profundiconus*? *hennigi* sp. nov.	3			3
Conidae spp.	8	19	7	34
**No. Specimens**	**85**	**129**	**145**	**359**
**No. Species**	15	14	16	28

Number of specimens of each species observed at locality stations TU 1422, 1215, and 1354. Counts include specimens questionably assigned to a given taxon. Total numbers of species observed at each station are minimal estimates because they do not include specimens that have not been identified (i.e., Conidae spp.). (Note: two specimens of *Conus symmetricus* [PRI 66175, TU 1354 and PRI 67707, TU 1215] that are included in the counts above are currently missing.)

**Genus *Profundiconus* Kuroda, 1956**

Type species: *Chelyconus profundorum* Kuroda, 1956.

***Profundiconus*? *hennigi* Hendricks sp. nov**.

urn:lsid:zoobank.org:act:9B5C69B8-01EF-474E-88D1-F70584C3FDA8

Fig. 4, S2 Table

**Fig 4:**
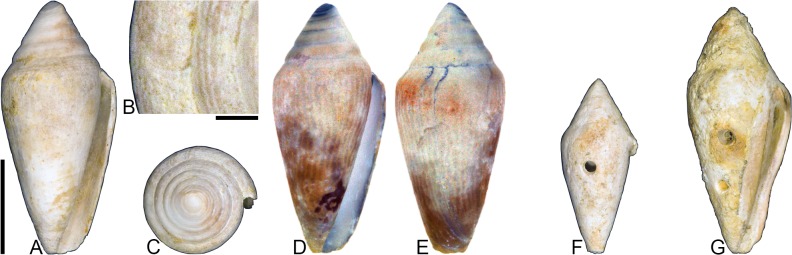
*Profundiconus*? *hennigi* Hendricks sp. nov. Specimens are from locality station TU 1422 (Cercado Fm.). (A-E) PRI 66164 (holotype), SL 25.5 mm; (F) PRI 67168 (paratype), SL 17.6 mm; (G) PRI 67167 (paratype), SL 24.9 mm. (D-E) are reversed images of the holotype photographed under UV light. Scale bar to the left of (A) is 1 cm and pertains to (A, C-G); (B) scale bar is 1 mm.

### Material examined

**Holotype**: PRI 66164. **Paratypes**: PRI 67167 and PRI 67168. All type specimens are from TU station 1422.

### Type locality and horizon

TU 1422: Arroyo Bellaco, Dominican Republic; upper Miocene Cercado Formation.

### Description

#### Shell size

Shell moderately small (largest observed specimen, PRI 66164, is 25.6 mm).

#### Last whorl

Ventricosely conical (RD 0.60–0.62, μ = 0.61; PMD 0.82–0.84, μ = 0.83; n = 2); outline slightly convex. Shoulder subangulate, smooth. Widest part of shell below shoulder. Aperture uniform in width from base to shoulder. Siphonal notch absent. Widely spaced spiral threads on anterior half that are sometimes beaded.

#### Spire whorls

Spire height high (RSH 0.27–0.30, μ = 0.29; n = 2); outline straight. Protoconch unknown. Early postnuclear whorls unknown; tubercles absent from preserved whorls. Sutural ramp convex, with at least 3 spiral threads. Subsutural flexure nearly symmetrical, depth approximately 0.9x width.

#### Coloration pattern

One pattern present. Pattern consists of wavy to nearly straight, typically non-branching thin axial streaks that in many cases extend from the base to the shoulder. Axial streaks on the last whorl appear to extend onto the sutural ramp.

### Etymology

Named for Willi Hennig (1913–1976), developer of cladistics.

### Remarks

The only co-occurring fossil species with an overall shell shape—particularly a high spire—that is similar to *Profundiconus*? *hennigi* is *Conus bellacoensis* sp. nov., but that species has a very different coloration pattern, has tubercles on most of the spire whorls, and also lacks spiral threads on its sutural ramp. There are no extant western Atlantic cone snails that have a shell morphology similar to *P*.? *hennigi*. Interestingly, however, *P*.? *hennigi* does bear some resemblance in shell form to a living Indo-Pacific cone species that Tucker and Tenorio [34] assigned to the genus *Profundiconus* Kuroda, 1956: *P*. *lani* Crandall, 1979, which was illustrated by Röckel et al. ([6], pl. 27, Figs. 10–12). Given the strong similarity in shell form between this new fossil species and *Profundiconus lani*, the same genus-level assignment is tentatively followed here (Puillandre et al. [1] also recognized *Profundiconus* as a distinct clade of cone snails and demonstrated that it is basal to all other cone snail clades). *Profundiconus*? *hennigi* is the only known representative of the genus in the Neogene of the Dominican Republic.

**Genus *Conasprella* Thiele, 1929**

Type species: *Conus pagoda* Kiener, 1847.

***Conasprella stenostoma* (Sowerby I, 1850)** [37]

Fig. 5

**Fig 5:**
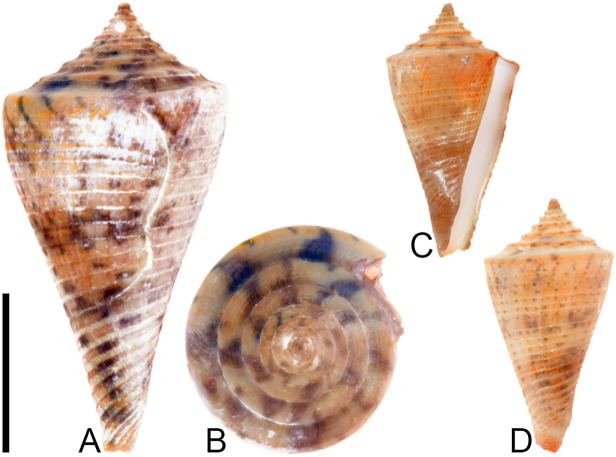
*Conasprella stenostoma* Sowerby I, 1850. Both specimens are from locality station TU 1215 (Gurabo Fm.). (A-B) PRI 66148, SL 28.1 mm; (C-D) PRI 67549, SL 16.0 mm. All are reversed images of specimens photographed under UV light. Scale bar is 1 cm and pertains to all images.

*Conus stenostoma* Sowerby I, 1850 [37], p. 44; Guppy, 1866 [38], p. 287, pl. 16, Fig. 2; Pilsbry, 1921 [39], p. 327–328, pl. 21, Fig. 1.

*Conus stenostomus* Sowerby I, Maury, 1917 [40], p. 203, pl. 6, Fig. 4.

*Kohniconus stenostoma* (Sowerby I), Tucker and Tenorio, 2009 [34], p. 146.

### Material examined

PRI 66148, PRI 67549 (TU station 1215).

### Coloration pattern

Two noninteracting patterns present. The primary (base) pattern consists of 3 discontinuous spiral bands that sometimes form blotches. The secondary pattern (observed here in PRI 67549) consists of about 20 spiral rows of dots; these sometimes coalesce near the shoulder to form short diagonal lines. The two patterns differ in the color of emitted light. Sutural ramp with blotches.

## Remarks

The distinctive shell shape and coloration pattern of *Conasprella stenostoma* allows it to be easily distinguished from co-occurring fossil species. Among extant western Atlantic species, it is most similar to *C*. *delessertii* Récluz, 1843, which was recently characterized by Kohn [8]. The coloration patterns of the two species are similar (e.g., see [8], pl. 24, Figs. 4–5). One notable difference is that spiral groves cover the last whorl of *C*. *stenostoma*, but these are restricted to the anterior third in *C*. *delessertii* [8]. While Tucker and Tenorio [34] assigned both *C*. *stenostoma* and *C*. *delessertii* to the genus *Kohniconus* Tucker and Tenorio, 2009, Puillandre et al. [1] found that *C*. *delessertii* did not group with other species that had been assigned to *Kohniconus*, which was treated as a subgenus of *Conasprella* Thiele, 1929 [1, 2]. Given its strong resemblance to *C*. *delessertii* and the taxonomic decision by Puillandre et al. [1, 2] to characterize the taxonomic status of *C*. *delessertii* as “incertae sedis” within *Conasprella*, *C*. *stenostoma* is not assigned to a subgenus at this time.

**Subgenus *Dalliconus* Tucker and Tenorio, 2009**

Type species: *Conus mcgintyi* Pilsbry, 1955.

***Conasprella* (*Dalliconus*) cf. *sauros* (Garcia, 2006)** [41]

Fig. 6

**Fig 6:**
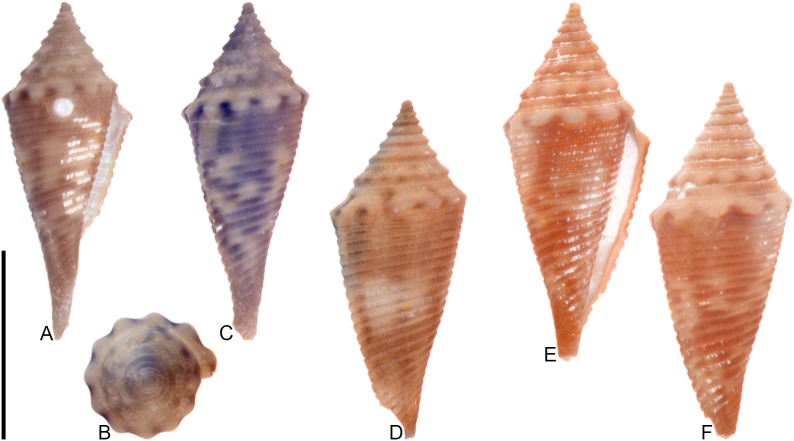
*Conasprella* (*Dalliconus*) cf. *sauros* Garcia, 2006. All specimens are from locality station TU 1215 (Gurabo Fm.). (A-C) PRI 67585, SL 17.8 mm; (D) PRI 67581, SL 17.9 mm; (E-F) PRI 67580, SL 18.9 mm. All are reversed images of specimens photographed under UV light. Scale bar is 1 cm and pertains to all images.

*Conus sauros* Garcia, 2006 [41], p. 71–74, Figs. 1–9, 16, 18, 21, 22; Kohn, 2014 [8], p. 126–128, pl. 22, Figs. 1–18.

*Conasprella* (*Dalliconus*) *sauros* (Garcia), Puillandre et al., 2015 [2], p. 5.

*Conus tortuosostriatus* Maury, 1917 [40], p. 205, pl. 6, Fig. 2; not Toula, 1911.

## Material examined

PRI 66136, PRI 67578–67591 (TU station 1215).

## Coloration pattern

Probably one pattern present. Pattern consists of irregularly-spaced spiral dashes that often coalesce to form axial streaks, blotches, or discontinuous spiral bands; spaces between shoulder tubercles heavily pigmented. Sutural ramp with occasional blotches.

## Remarks

This small, high-spired species is common in some Dominican fossil localities, but the name that should be applied to it is not clear. Maury [40] figured a specimen of this species (PRI 28606) and called it *Conus tortuosostriatus* Toula, 1911, a species likely described from the Miocene Gatun Formation of the Canal Zone, Panama [42]. Woodring [42] showed that *C*. *tortuosostriatus* is a different species and referred Maury’s [40] specimen to *C*. *gabbi* Pilsbry and Johnson, 1917. The type of *C*. *gabbi* (ANSP 2553) was observed (see Pilsbry, 1921 [39], pl. 21, Fig. 9) and it appears to be a different species: it is much larger (SL 42.5 mm) and the posterior half of its last whorl is convex in profile, while it is nearly straight in this species. The type of *C*. *gracilissimus* Guppy, 1866—a taxon originally described from Jamaica—is similar to this species, but bears tubercles on all of its spire whorls, which is not a characteristic of the species treated here, which has weak tubercles on the first postnuclear whorl and on the final spire whorls, but not in-between. This species is similar to the extant western Atlantic species *C*. *sauros* (Garcia, 2006)—which was recently characterized by Kohn [8]—and placed in the genus *Conasprella* and subgenus *Dalliconus* Tucker and Tenorio, 2009 by Puillandre et al. [2]. Once again, however, the fossil species differs by not having tubercles on all of its spire whorls, which is a feature of *Conasprella sauros*. Determination of whether this fossil form, which has been known at least since Maury [40], requires a new name will require additional research; until then, it is simply compared to the modern species as *Conasprella* cf. *sauros*.

**Subgenus *Ximeniconus* Emerson and Old, 1962**

Type species: *Conus ximenes* Gray, 1839.

***Conasprella* (*Ximeniconus*) *cercadensis* (Maury, 1917)** [40]

Fig. 7A-E

**Fig 7:**
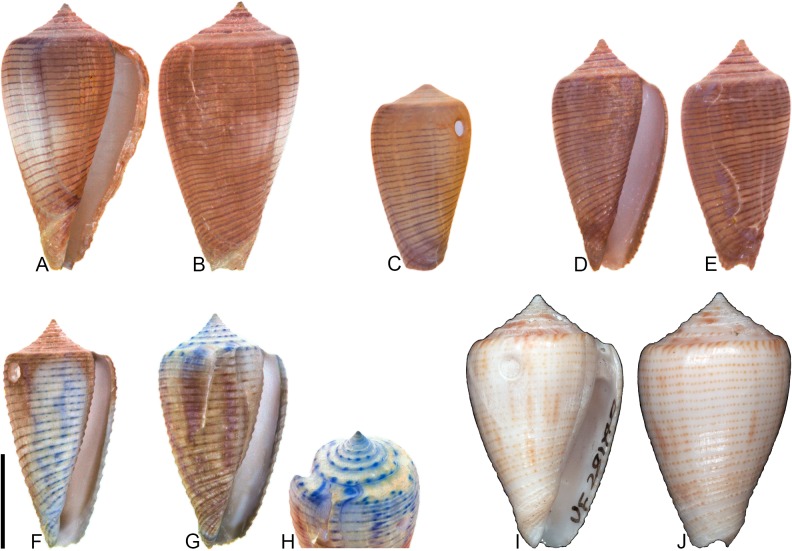
*Conasprella* (*Ximeniconus*) *cercadensis* Maury, 1917 (A-E), *Conasprella* (*Ximeniconus*) *kitteredgei* Maury, 1917 (F-H), and *Conasprella* (*Ximeniconus*) *puncticulata* (Hwass in Bruguière, 1792) (I-J). Fossil specimens are from locality stations TU 1422 (Cercado Fm.), TU 1215 (Gurabo Fm.), and TU 1354 (Gurabo Fm.); modern *C*. *puncticulata* is from Bateau Bay, Trinidad and Tabago. (A-B) PRI 66153, TU 1422, SL 27.3 mm; (C) PRI 66150, TU 1215, SL 18.8 mm; (D-E) PRI 67185, TU 1422, SL 23.6 mm; (F) PRI 67546, TU 1354, SL 22.9 mm; (G-H) PRI 66183, TU 1354, SL 23.5 mm; (I-J) UF 281140, SL 25.5 mm. (A-H) are reversed images photographed under UV light. Scale bar is 1 cm and pertains to all images.

*Conus cercadensis* Maury, 1917 [40], p. 207–208, pl. 7, Fig. 4; Pilsbry, 1921 [39], p. 332, pl. 20, Fig. 10.

*Perplexiconus cercadensis* (Maury), Tucker and Tenorio, 2009 [34], p. 149.

## Material examined

**Holotype**: PRI 28613. **Other specimens**: PRI 66153, PRI 67185, PRI 67235, and PRI 67237 (TU station 1422); PRI 66150 (TU station 1215).

## Coloration pattern

One pattern present. Pattern consists of about 30–40 spiral lines, extending from base to shoulder; these coincide with the raised posterior edges of spiral ribs on the anterior half of the last whorl. Sutural ramp unpigmented.

## Remarks

*Conasprella cercadensis* and *C*. *kitteredgei* (Maury, 1917) have similar overall shell morphologies, but their preserved coloration patterns exhibit subtle differences: *C*. *kitteredgei* shows evidence of axial streaks and spiral dashes on the last whorl, in addition to dots on the sutural ramp, none of which are observed in *C*. *cercadensis*. Both taxa appear to be closely related to the Recent western Atlantic species *C*. *puncticulata* (Hwass in Bruguière, 1792) (Fig. 7I,J), which—with its axial streaks and rows of discontinuous spiral lines—has a coloration pattern that is most similar to that of *C*. *kitteredgei*. *Conasprella puncticulata* is the sister species of *C*. *perplexa* (Sowerby II, 1857) [1], a Recent eastern Pacific taxon. Tucker and Tenorio [34] assigned both *C*. *cercadensis* and *C*. *kitteredgei* to the genus *Perplexiconus* Tucker and Tenorio, 2009, which was considered by Puillandre et al. [2] a synonym of *Ximeniconus* Emerson and Old, 1962, a name that they applied at the subgeneric level and which is followed here.

***Conasprella* (*Ximeniconus*) *kitteredgei* (Maury, 1917)** [40]

Fig. 7F-H

*Conus kitteredgei* Maury, 1917 [40], pl. 7, Fig. 6.

*Perplexiconus kitteredgei* (Maury), Tucker and Tenorio, 2009 [34], p. 149.

## Material examined

**Syntype**: PRI 28615. **Other specimens**: PRI 66183, PRI 66187, PRI 67545, and PRI 67546 (TU station 1354).

## Coloration pattern

Two weakly interacting patterns present. The primary (base) pattern consists of axial streaks that extend across much of the last whorl. Secondary pattern consists of about 20–22 discontinuous spiral lines (forming dashes) that extend from base to shoulder; these coincide with the raised posterior edges of spiral ribs on the entirety of the last whorl. The secondary pattern appears to be more intense in areas where it intersects the primary pattern. The two patterns differ slightly in the color of emitted light. Sutural ramp with dots on the abaxial margin.

## Remarks

See remarks for *C*. *cercadensis* for a discussion of how its coloration pattern differs from *C*. *kitteredgei*, as well as a comparison with modern taxa.

*Conasprella* (*Ximeniconus*) *ageri* Hendricks sp. nov.

urn:lsid:zoobank.org:act:5AEE94B0-AC55-4F8F-80EF-E5462E70BC4A

Fig. 8A-P, S2 Table

**Fig 8:**
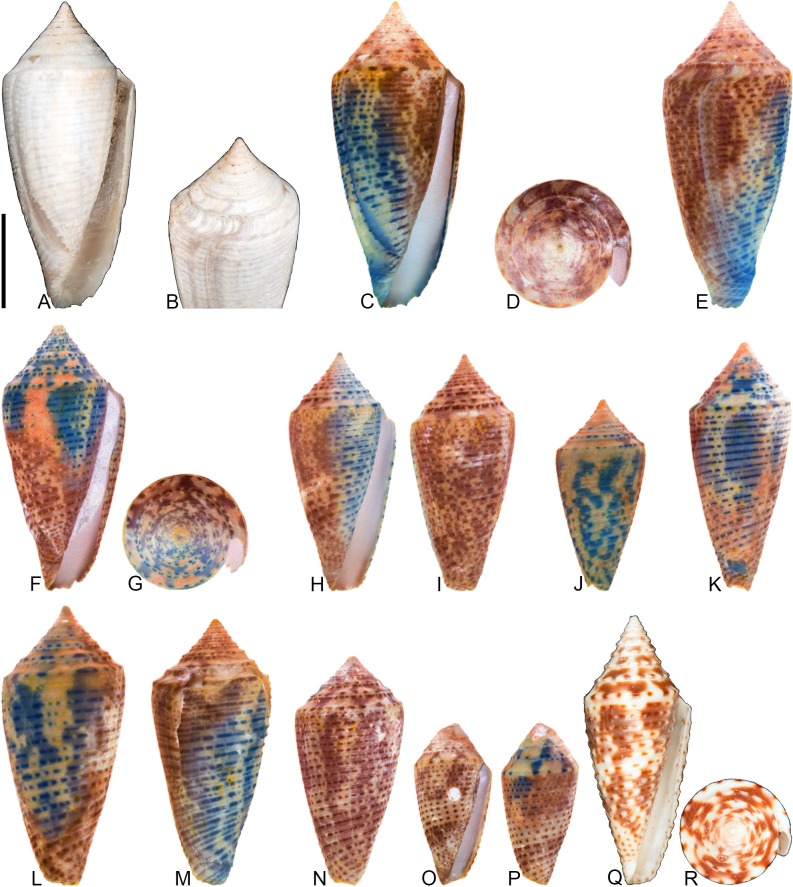
*Conasprella* (*Ximeniconus*) *ageri* sp. nov. (A-P) and *Conasprella* (*Ximeniconus*) *tornata* (Sowerby II, 1833) (Q-R). Fossil specimens are from locality stations TU 1422 (Cercado Fm.) and TU 1354 (Gurabo Fm.); modern *C*. *tornata* is from Tortola Island, Bay of Panama. (A-E) PRI 67515, TU 1354, SL 30.8 mm; (F-G) PRI 67520, TU 1354, SL 26.9 mm; (H-I) PRI 67510, TU 1354, SL 24.1 mm; (J) PRI 67532, TU 1354, SL 19.4 mm; (K) PRI 67512, TU 1354, SL 25.1 mm; (L) PRI 66171, TU 1354, SL 28.0 mm; (M) PRI 67523, TU 1354, SL 26.8 mm; (N) PRI 67527, TU 1354, SL 23.2 mm; (O-P) PRI 67231, TU 1422, SL 16.4 mm; (Q-R) UF 355879, SL 28.4 mm. Scale bar is 1 cm and pertains to all images.

## Material examined

**Holotype**: PRI 67515. **Paratypes**: PRI 66171, PRI 67510–67514, PRI 67516–67536. All type specimens are from TU station 1354. **Other specimens**: PRI 67231–67232 (TU station 1422).

## Type locality and horizon

TU 1354: Cañada de Zamba, Dominican Republic; lower Pliocene Gurabo Formation.

## Description

### Shell size

Shell moderately small (largest observed specimen, PRI 67514, is 32.5 mm).

### Last whorl

Typically conical, sometimes ventricosely conical (RD 0.54–0.61, μ = 0.57; PMD 0.83–0.89, μ = 0.86; n = 17); outline slightly convex, except at anterior quarter, which is slightly concave. Shoulder sharply angulate, smooth. Widest part of shell just below shoulder. Aperture slightly wider at base than near shoulder. Siphonal notch absent; anterior-most end of lip sometimes extends slightly beyond columella. Incised spiral grooves on anterior half that diminish towards the shoulder; pustules sometimes present on adapical surfaces of intervening ribs (e.g., PRI 67510).

### Spire whorls

Spire height moderate (RSH 0.18–0.23, μ = 0.20; n = 17); outline slightly concave. Protoconch diameter 0.7–0.8 mm (based on PRI 67510, PRI 67511, and PRI 67518), number of whorls unknown. Tubercles absent from all spire whorls. Sutural ramp flat to concave on early whorls, concave in later whorls; spiral ornamentation absent, but growth lines prominent across sutural ramp. Subsutural flexure symmetrical and square-shaped to slightly curved abaxially; depth 1.5–2.3x width.

### Coloration pattern

Two noninteracting patterns present. The primary (base) pattern consists of axially-arranged, irregularly-shaped blotches of variable width. The secondary pattern typically consists of about 20–30 spiral rows of dots or dashes. The two patterns differ in the color of emitted light. Sutural ramp with blotches.

### Etymology

Named in honor of paleontologist Derek V. Ager, author of “The Nature of the Stratigraphical Record” [43].

### Remarks

A specimen of cone snail (NHMUK PI BM 64050) remarkably similar to *Conasprella ageri* is present in collections at the NHMUK associated with Guppy’s [38] report on fossil mollusks from Jamaica (photographs of the specimen may be viewed online through the NHMUK Palaeontology Collection Database: http://www.nhm.ac.uk/research-curation/scientific-resources/collections/palaeontological-collections/palaeontology-specimen-database/index.php). NHMUK PI BM 64050 is labeled as “*Conus solidus*,” a very differently shaped species that is synonymous with *Conus recognitus* Guppy, 1867 (this was recognized by Maury [40], Pilsbry [39], and Woodring [42]). It does not appear that the distinctive morphology of NHMUK PI BM 64050 was ever formally described by Guppy or other workers. It is likely a specimen of *C*. *ageri*, as its shell characteristics are consistent with the new species (it is not known at the present time, however, whether it bears a preserved coloration pattern visible under UV light). Two poorly preserved shells—PRI 67231 and PRI 67232—are from TU station 1422 and may be additional specimens of *C*. *ageri*. PRI 67231 has a well-preserved coloration pattern that is consistent with *C*. *ageri*, except that the primary pattern consists of two nearly continuous spiral bands that separate an unpigmented region in the middle of the last whorl (see Fig. 8O,P). The specimens of *Conus granozonatus* Guppy, 1866 figured by Woodring [44] (from Jamaica) and holotype of *Conus trisculptus* Pilsbry and Johnson, 1917 (ANSP 2567) (from the Dominican Republic) are very similar in shell form to *C*. *ageri*, but both species show evidence of tubercles on the shoulder, which are not present on *C*. *ageri*.

Among extant species, *C*. *ageri* is most similar in shell morphology and coloration pattern to the eastern Pacific species *Conasprella tornata* (Sowerby II, 1833) (Fig. 8Q,R). *Conasprella tornata* was assigned by Puillandre et al. [2] to the subgenus *Ximeniconus* and this assignment is also followed here for *C*. *ageri*.

**Genus *Conus* Linnaeus, 1758**

Type species: *Conus marmoreus* Linnaeus, 1758.

***Conus symmetricus* Sowerby I, 1850** [37]

Fig. 9

**Fig 9:**
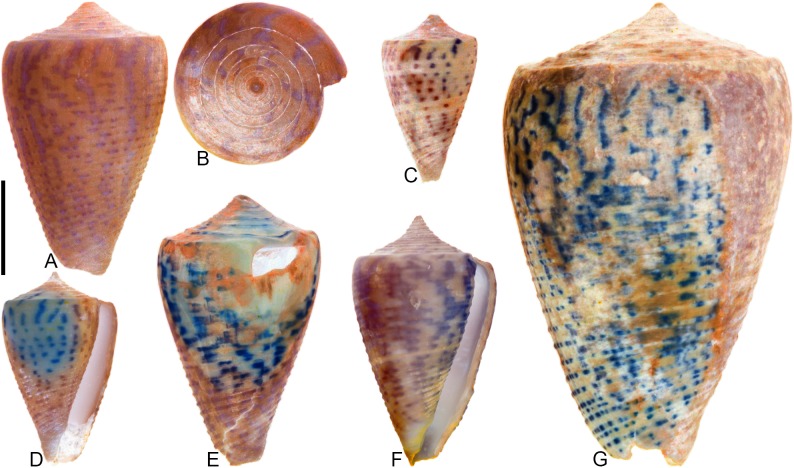
*Conus symmetricus* Sowerby I, 1850. Specimens are from locality stations TU 1422 (Cercado Fm.), TU 1215 (Gurabo Fm.), and TU 1354 (Gurabo Fm.). (A-B) PRI 67665, TU 1215, SL 27.3 mm; (C) PRI 67190, TU 1422, SL 17.1 mm; (D) PRI 67192, TU 1422, SL 19.5 mm; (E) PRI 67191, TU 1422, SL 27.4 mm; (F) PRI 67675, TU 1354, SL 26.2 mm; (G) PRI 66155, TU 1422, SL 46.1 mm. All are reversed images of specimens photographed under UV light. Scale bar is 1 cm and pertains to all images.

*Conus symmetricus* Sowerby I, 1850 [37], p. 44, pl. 9, Fig. 1; Maury, 1917 [40], pl. 7, Fig. 7, 7A; Pilsbry, 1921 [39], pl. 20, Fig. 2, 2A, 2B.

*Conus* (*Leptoconus*) *symmetricus* Sowerby I, Pflug, 1961 [45], p. 63–64, pl. 18, Figs. 1–11.

*Purpuriconus symmetricus* (Sowerby I), Tucker and Tenorio, 2009 [34], p. 116.

### Material examined

**Lectotype**: NHMUK PI BM G 83969 (designated by Pflug [45]). **Other specimens**. PRI 66155, PRI 67187–67188, PRI 67190–67192, PRI 67228–67229 (TU station 1422); PRI 66135, PRI 67665, PRI 67701–67741 (TU station 1215); PRI 66170, PRI 66175, PRI 67672–67699 (TU station 1354).

### Coloration pattern

While otherwise consistent in shell morphology, specimens of *C*. *symmetricus* show a wide range of variability in coloration pattern. Two general types of observed coloration pattern are described here.

Type 1 (see Fig. 9A,B,F,G). Two noninteracting patterns present. The primary (base) pattern consists of irregularly-shaped axial streaks or spots that cover most of the last whorl. The secondary pattern consists of about 17–20 spiral rows of dots that correspond with beaded spiral ribs on the basal two-thirds of the last whorl. The two patterns differ slightly in the color of emitted light. Sutural ramp with radial streaks.

Type 2 (see Fig. 9C,D, questionably E). Two noninteracting patterns present. The primary (base) pattern consists of three weakly-pigmented, discontinuous spiral bands. The secondary pattern consists of about 20 spiral rows of dots, which sometimes coalesce near the shoulder to form axial streaks. The two patterns differ slightly in the color of emitted light. Sutural ramp with radial streaks.

### Remarks

*Conus symmetricus*—which is a common species, especially in the Gurabo Fm.—has a very distinctive shell shape that is not easily confused with co-occurring species. Its relationship to extant species is not clear. Petuch [46] stated that *C*. *tristensis* Petuch, 1987 “probably represents the last living member of the *C*. *symmetricus-dominguensis* species complex” ([46], p. 113) (also see [8], p. 272). Kohn [8] considered *C*. *tristensis* a synonym, albeit somewhat tentatively, of *C*. *cancellatus* Hwass in Bruguière, 1792, which was assigned to the subgenus *Dauciconus* by Puillandre et al. [1, 2]. While many specimens of *C*. *symmetricus* have an overall shell shape that is somewhat similar to that of the holotype of *C*. *tristensis* (see [8], pl. 69, Figs. 6, 7), specimens of *C*. *symmetricus* always have beaded ribs on the last whorl, which were not reported by Kohn [8] in his recent description of *C*. *cancellatus*. Additionally, shells of *C*. *symmetricus* are widest below the shoulder, but specimens of *C*. *cancellatus* are usually widest at the shoulder. Finally, coloration patterns similar to those described above for *C*. *symmetricus* are not present in any known specimens of *C*. *cancellatus*, which are often—though not always (see [8])—unpigmented. Tucker and Tenorio [34] assigned *C*. *symmetricus* to the genus *Purpuriconus* da Motta, 1991, which they defined in part as having “only 1 cord that may not persist on the whorl tops” (p. 115); *C*. *symmetricus*, however, has several spiral threads on the sutural ramp (e.g., Fig. 9B; Pflug [45], pl. 18, Figs. 5, 10). Because its phylogenetic position cannot be resolved at this time, *C*. *symmetricus* is not assigned to a subgenus.

***Conus anningae* Hendricks sp. nov**.

urn:lsid:zoobank.org:act:FC37256A-4348-4916-A241-F4BDBB4F4552

Fig. 10, S2 Table

**Fig 10:**
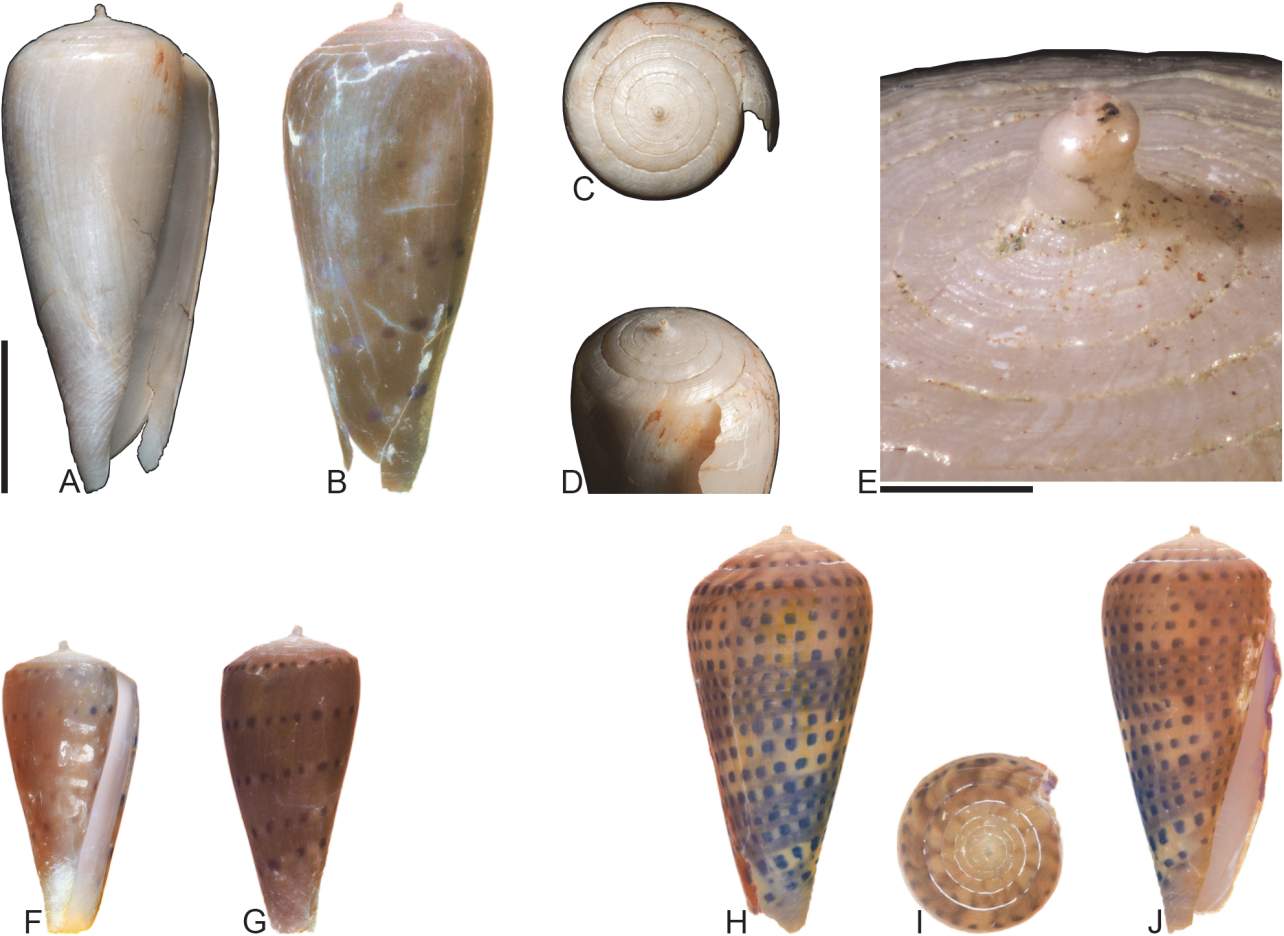
*Conus anningae* Hendricks sp. nov. All specimens are from locality station TU 1215 (Gurabo Fm.). (A-E) PRI 66141 (holotype), SL 30.3 mm; (F-G) PRI 67567 (paratype), SL 19.2 mm; (H-J) PRI 67569 (paratype), SL 25.2 mm. (B, F-J) are reversed images of specimens photographed under UV light. Scale bar to left of (A) is 1 cm and pertains to images (A-D and F-J); scale bar below (E) is 1 mm and pertains to that image. (E) is a focal stacked image constructed from 10 separate images using Helicon Focus software (http://www.heliconsoft.com/).

### Material examined

**Holotype**: PRI 66141. **Paratypes**: PRI 67567, PRI 67569. All type specimens are from TU station 1215.

### Type locality and horizon

TU 1215: Río Gurabo, Dominican Republic; lower Pliocene Gurabo Formation.

### Description

#### Shell size

Shell moderately small (largest observed specimen, PRI 66141, is 30.3 mm).

#### Last whorl

Conoid-cylindrical (RD 0.50–0.53, μ = 0.52; PMD 0.82–0.85, μ = 0.84; n = 3); outline slightly convex, except at anterior quarter, which is slightly concave. Shoulder subangulate; smooth. Widest part of shell below shoulder. Aperture slightly wider at base than near shoulder. Siphonal notch absent. Spiral threads on anterior third.

#### Spire whorls

Spire height low (RSH 0.10–0.11, μ = 0.10; n = 3); outline sigmoidal. Protoconch with >3 whorls (based on PRI 66141), diameter 0.7–0.8 mm (based on all three known specimens); protrudes above subsequent whorls. Tubercles present on first 0.5–2 postnuclear whorls, becoming weakly undulate on following several whorls before diminishing. Sutural ramp concave on early whorls, convex on later whorls, with multiple spiral threads. Subsutural flexure asymmetrical, depth about equal to width.

#### Coloration pattern

Two weakly interacting patterns present. PRI 66141 (holotype; Fig. 10A-E) and PRI 67567 (Fig. 10F,G) appear to have a solidly pigmented primary (base) pattern that extends from the anterior end to the shoulder; in PRI 67569 (Fig. 10H-J), this primary pattern is restricted to two pigmented bands that each cover about one-fifth of the last whorl. The secondary pattern consists of 5 or at least 6 (PRI 67567 and PRI 66141, respectively) to as many as 19 (PRI 67569) spiral rows of square-shaped dots. Interaction between the two patterns occurs when the spiral dots intersect the base pattern, resulting in unpigmented spaces between the spiral dots (e.g., see anterior band on Fig. 10H). The two patterns differ in the color of emitted light. Sutural ramp with radial streaks that correspond with the shape of the subsutural flexure.

### Etymology

Named for Mary Anning (1799–1847) in honor of the important fossil discoveries that she made at Lyme Regis, England. Anning is the person referred to in Terry Sullivan’s famous tongue-twister “she sells seashells on the seashore.”

### Remarks

The preserved coloration pattern of paratype PRI 67569 (Fig. 10H-J) differs significantly from that of the other two known specimens in the number of spiral rows of dots (see above), but this shell is otherwise consistent in other aspects of shell form. It is assumed, therefore, that these differences in coloration pattern amount to intraspecific variation and that all three specimens belong to the same species. Comparison with Kohn [8] reveals that *Conus anningae* is not similar in shell morphology to any extant western Atlantic cone snail species, nor is it similar to any other known extant or fossil species. It may therefore represent an extinct lineage of Conidae.

***Conus lyelli* Hendricks sp. nov**.

urn:lsid:zoobank.org:act:8D6708E0-9262-4CA2-B03D-39A4EFB5081B

**Fig 11:**
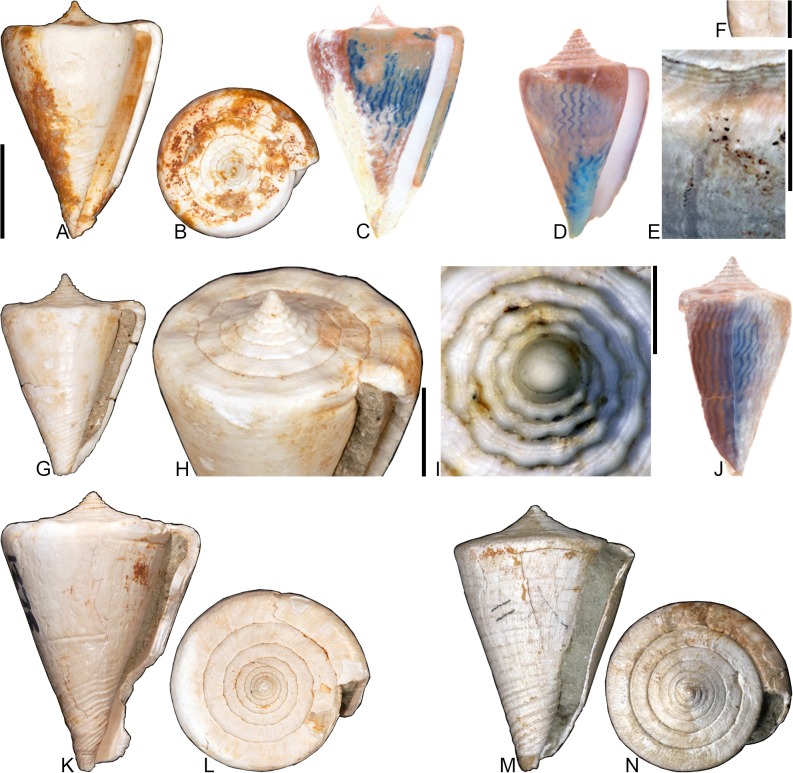
*Conus lyelli* Hendricks sp. nov. (A-L) and *Conus xenicus* Pilsbry and Johnson, 1917 (M-N). Specimens of *C*. *lyelli* are from locality stations TU 1422 (Cercado Fm.), TU 1278 (Gurabo Fm.), and TU 1354 (Gurabo Fm.); the specimen label associated with the holotype of *C*. *xenicus* (M-N) reports the locality as “S. Domingo.” (A-C) PRI 66156 (holotype), TU 1422, SL 23.7 mm; (D-F) PRI 67165 (paratype), TU 1354, SL 21.2 mm; (G-I) PRI 66189 (paratype), TU 1422, SL 20.3 mm; (J) PRI 66184 (paratype), TU 1354, SL 22.3 mm; (K-L) PRI 66115 (paratype), TU 1278, SL 29.4 mm (estimated from digital image); (M-N) ANSP 2575 (holotype), SL 28.3 mm (estimated from digital image). (C-D, J) are reversed images of specimens photographed under UV light. (I) is a focal stacked image constructed from 9 separate images using Helicon Focus software (http://www.heliconsoft.com/). Scale bar to the left of (A) is 1 cm and pertains to (A-D, G, J-N); (E, H) scale bars are 5 mm; (F) scale bar is 2 mm; (I) scale bar is 1 mm.

Fig. 11A-L, S2 Table

### Material examined

**Holotype**: PRI 66156 (TU station 1422). **Paratypes**: PRI 66189–66192 (TU station 1422); PRI 66184 and 67165 (TU station 1354); PRI 66115 (TU station 1278).

### Type locality and horizon

TU 1422: Arroyo Bellaco, Dominican Republic; upper Miocene Cercado Formation.

### Other localities and horizons

TU 1354: Cañada de Zamba, Dominican Republic; lower Pliocene Gurabo Formation. TU 1278: Río Gurabo, Dominican Republic; lower Pliocene Gurabo Formation.

### Description

#### Shell size

Shell moderately small (largest observed complete specimen, PRI 66156, is 23.7 mm; a fragment of a larger specimen, PRI 66192, with MD 22.8 mm is also known, suggesting a complete shell size of about 35 mm).

#### Last whorl

Broadly conical (RD 0.70–0.75, μ = 0.73; PMD 0.89–0.93, μ = 0.91; n = 3); outline slightly sigmoidal. Shoulder subangulate, with undulations. Widest point of shell at shoulder. Aperture uniform in width from base to shoulder. Siphonal notch absent. Spiral threads on anterior half, diminishing towards shoulder.

#### Spire whorls

Spire height low to moderate (RSH 0.09–0.17, μ = 0.15; n = 3), with later whorls depressed beneath shoulder in some specimens; outline concave. Protoconch with 2.2 whorls, diameter 0.8 mm (based on PRI 66189). Tubercles present on first several postnuclear whorls, becoming posterior-pointing shoulder undulations in later whorls. Sutural ramp slightly concave to flat, with 3–6 fine spiral threads. Subsutural flexure symmetrical, depth about 0.6–0.8x width.

#### Coloration pattern

One pattern present. Pattern consists of slightly jagged to saw-toothed, non-branching thin axial streaks that in many cases extend from the base to the shoulder. There is no evidence of a coloration pattern on the spire whorls.

### Etymology

Named for the great geologist Charles Lyell (1797–1875), who popularized the concepts of uniformitarianism and the antiquity of the earth.

### Remarks

*Conus lyelli* is most similar in shell shape to *Conus xenicus* Pilsbry and Johnson, 1917 (Fig. 11M,N), which is also known from the Neogene of the Dominican Republic. An important difference is that *C*. *lyelli* possesses tubercles or shoulder undulations on all postnuclear whorls, while these features are absent in the holotype of *C*. *xenicus* (ANSP 2575). Additionally, the shoulder in *C*. *xenicus* is angulate and this species also has much more prominent spiral threads on its sutural ramp. As detailed locality information is not available for *C*. *xenicus*, it is not known whether it was contemporaneous with *C*. *lyelli*. Tucker and Tenorio [34] assigned *C*. *xenicus* to the genus *Jaspidiconus* Petuch, 2003, but it does not have a shell like extant species that have been assigned to that clade (note that Puillandre et al. [2] recognized *Jaspidiconus* as synonym of *Conasprella*, subgenus *Ximeniconus*). *Conus ornatus* of Maury, 1917 (not of Röding, which in turn is a junior synonym of *C*. *generalis* Linnaeus, 1767; see Röckel et al. [6]) and *C*. *williamgabbi* Maury, 1917 are also somewhat similar to *C*. *lyelli*, both having broad shells with low spires. Like *C*. *xenicus*, *C*. *ornatus* differs from the new species in its lack of tuberculate postnuclear whorls. *Conus williamgabbi* has an asymmetrical, deep subsutural flexure and is much larger than the new species. *Conus lyelli* is not similar to any known extant species, suggesting that it, along with *C*. *xenicus*, may be members of an extinct clade of cone snails.

**Subgenus *Atlanticonus* Petuch and Sargent, 2012**

Type species: *Conus granulatus* Linnaeus, 1758.

***Conus* (*Atlanticonus*?) *olssoni* (Maury, 1917)** [40]

Fig. 12A-I

**Fig 12:**
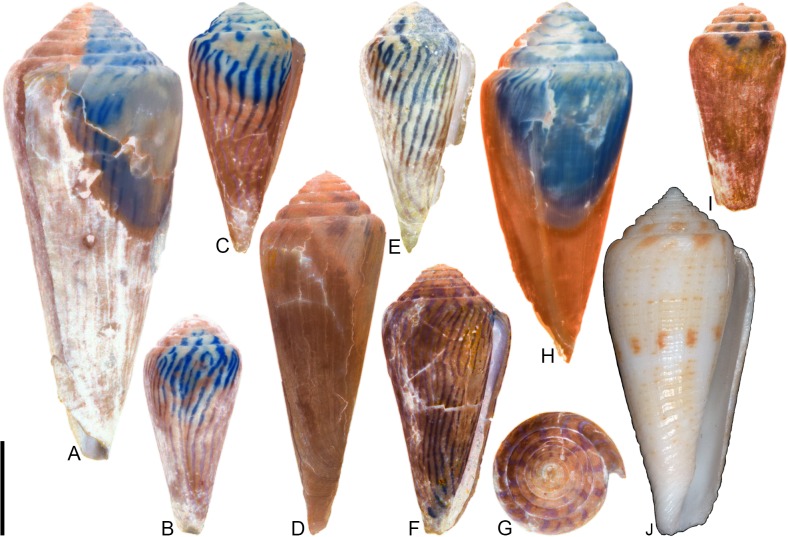
*Conus olssoni* Maury, 1917 (A-I) and *Conus granulatus* Linnaeus, 1758 (J). Fossil specimens are from locality stations TU 1422 (Cercado Fm.), TU 1215 (Gurabo Fm.), and TU 1354 (Gurabo Fm.); modern *C*. *granulatus* is from Isla Payardi, Bahia las Minas, Panama. (A) PRI 67198, TU 1422, SL 46.4 mm; (B) PRI 67203, TU 1422, SL 22.4 mm; (C) PRI 67199, TU 1422, SL 25.3 mm; (D) PRI 66137, TU 1215, SL 36.8 mm; (E) PRI 67204, TU 1422, SL 25.4 mm; (F-G) PRI 67200, TU 1422, SL 27.4 mm; (H) PRI 67245, TU 1354, SL 36.7 mm; (I) PRI 67598, TU 1215, SL 21.1 mm; (J) UF 329263–1, SL 35.0 mm. (A-I) are reversed images photographed under UV light. Scale bar is 1 cm and pertains to all images.

*Conus olssoni* Maury, 1917 [40], p. 207, pl. 7, Fig. 3.

*Dauciconus olssoni* (Maury), Tucker and Tenorio, 2009 [34], p. 89.

### Material examined

**Holotype**: PRI 28612. **Other specimens**: PRI 66157, PRI 67198–67206 (TU station 1422); PRI 66137, PRI 67592–67604 (TU station 1215); PRI 66180, PRI 67242, PRI 67244–67251 (TU station 1354).

### Coloration pattern

One pattern present. Pattern consists of slightly wavy to nearly straight, sometimes branching axial streaks that often extend from the base to the shoulder; these become finer in width as shell size increases. In some specimens (e.g., PRI 66137, Fig. 12D and PRI 67598, Fig. 12I), pigmented blotches occur at the shoulder; it is not clear how these relate to the principle pattern. The last whorl pattern extends over the shoulder onto the sutural ramp.

### Remarks

*Conus olssoni* has a distinctive shell shape that is not easily confused with co-occurring species. It does, however, bear some similarity in shell form to *Conus granulatus* Linnaeus, 1758 (Fig. 12J), an extant western Atlantic species recently characterized by Kohn [8]. In particular, both species have similarly shaped last whorls and stepped spire whorls. While some specimens of *C*. *granulatus* have faint axial streaks on the last whorl (e.g., [8], pl. 2, Figs. 10–11), these are much more pronounced in *C*. *olssoni*. Additionally, *C*. *granulatus* possesses spiral rows of ribs and pigmented dots on the last whorl; these were not observed in *C*. *olssoni*.

Tucker and Tenorio [34] placed *C*. *olssoni* in the genus *Dauciconus* Cotton, 1945. Given the similarities in shell shape that it shares with *C*. *granulatus*, however, it is instead tentatively placed in the subgenus *Atlanticonus* Petuch and Sargent, 2012, following the designation for *C*. *granulatus* by Puillandre et al. [2]. It is worth mentioning that none of the four extant species assigned to the subgenus *Atlanticonus* by Puillandre et al. [2] have been subjected to phylogenetic analysis; thus, this clade still requires verification.

***Conus* (*Atlanticonus*?) *franklinae* Hendricks sp. nov**.

urn:lsid:zoobank.org:act:3E38EC90-8C86-45AB-8DEC-20927DB90FFE

Fig. 13, S2 Table

**Fig 13:**
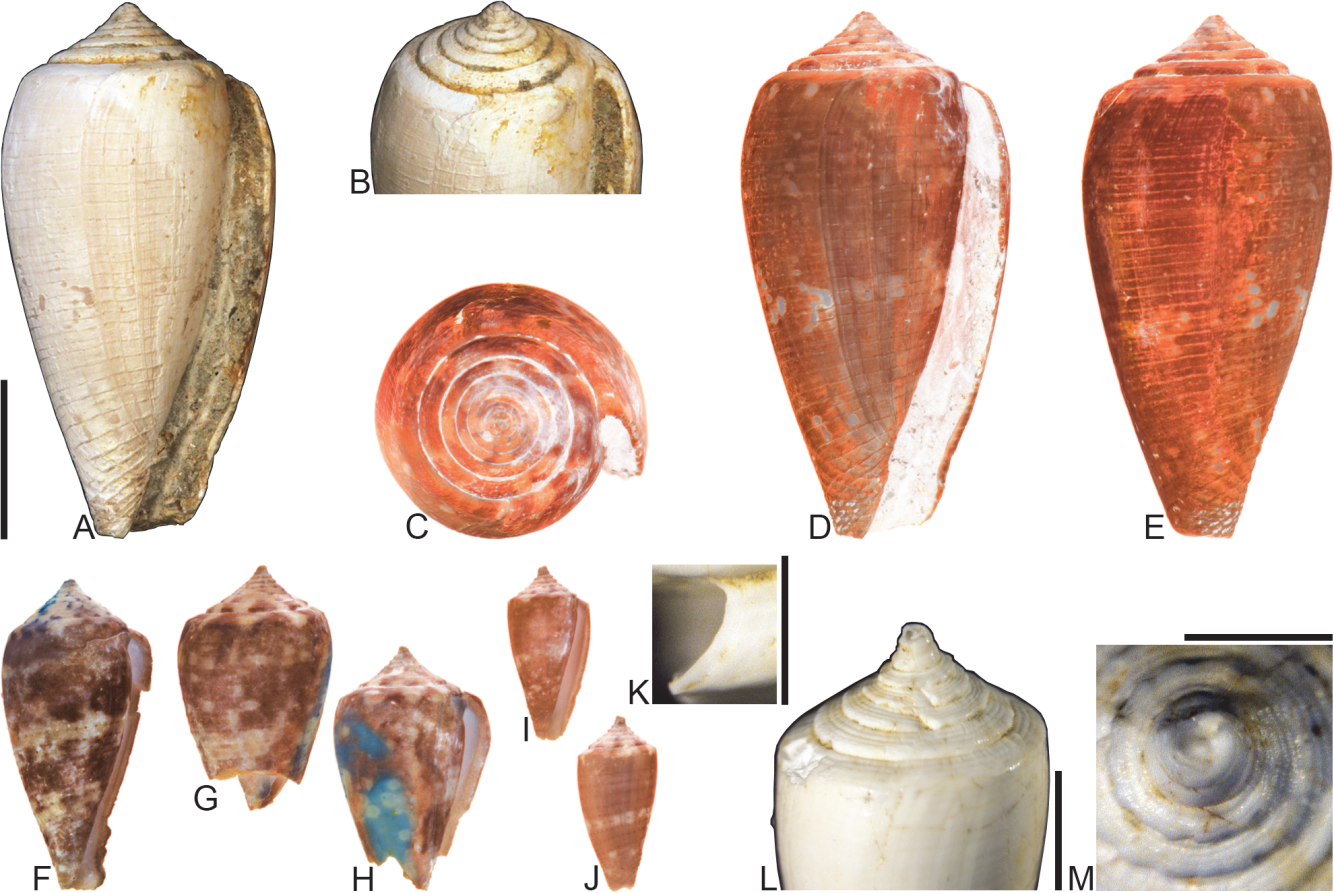
*Conus* (*Atlanticonus*?) *franklinae* Hendricks sp. nov. Specimens are from locality stations TU 1422 (Cercado Fm.) and TU 1215 (Gurabo Fm.). (A-E) PRI 66142 (holotype), TU 1215, SL 32.7 mm; (F) PRI 67227 (paratype), TU 1422, SL 18.8 mm; (G-H) PRI 67226 (paratype), TU 1422, SL 14.7 mm; (I-M) PRI 67230 (paratype), TU 1422, SL 10.5 mm. (C-J) are reversed images of specimens photographed under UV light. Scale bar to the left of (A) is 1 cm and pertains to (A-J); (K, M) scale bars are 1 mm; (L) scale bar is 2 mm.

### Material examined

**Holotype**: PRI 66142 (TU station 1215). **Paratypes**: PRI 67226, PRI 67227, and PRI 67230 (TU station 1422).

### Type locality and horizon

TU 1215: Río Gurabo, Dominican Republic; lower Pliocene Gurabo Formation.

### Other locality and horizon

TU 1422: Arroyo Bellaco, Dominican Republic; upper Miocene Cercado Formation.

### Description

#### Shell size

Shell moderately small (largest observed specimen, PRI 66142, is 32.7 mm).

#### Last whorl

Ventricosely conical (RD 0.58–0.59, μ = 0.58; PMD 0.79–0.83, μ = 0.81; n = 3); outline convex, except at anterior quarter, which is slightly concave. Shoulder subangulate, smooth. Widest part of shell below shoulder. Aperture uniform in width from base to shoulder. Siphonal notch present. Fine spiral threads on anterior half of juvenile specimens, extending to shoulder in mature specimens.

#### Spire whorls

Spire height moderate (RSH 0.13–0.19, μ = 0.16; n = 3); outline sigmoidal. Protoconch multispiral, diameter 0.8 mm (based on partial remains on PRI 67230). Tubercles present on first 2 postnuclear whorls. Sutural ramp slightly concave to flat on early whorls, convex on later whorls, with several spiral threads. Subsutural flexure asymmetrical, depth approximately 0.5–0.9x width.

#### Coloration pattern

Two noninteracting patterns present. The primary (base) pattern consists of a solid to blotchy ground pattern that is interrupted by 2–4 spiral rows of inconsistently arranged, unpigmented dots. The secondary pattern consists—at least in juvenile specimens—of at least 10 spiral rows of dots. The two patterns differ slightly in the color of emitted light. Sutural ramp with blotches that appear to be extensions of the primary last whorl pattern.

### Etymology

Named in honor of Rosalind Franklin (1920–1958) for the important role that she played in discovering the structure of DNA.

### Remarks

The shell morphology and coloration pattern of *Conus franklinae* is somewhat similar to that of *C*. *zambaensis* sp. nov. (see below). The two species do not overlap, however, in their positions of maximum diameter (PMD 0.79–0.83 in *C*. *franklinae*, 0.84–0.88 in *C*. *zambaensis*) or relative spire heights (RSH 0.13–0.19 in *C*. *franklinae*, 0.06–0.09 in *C*. *zambaensis*). Additionally, the subsutural flexure in *C*. *franklinae* is asymmetrical, while that of *C*. *zambaensis* is nearly symmetrical and deeper. *Conus franklinae* bears strong similarity to the extant western Atlantic species *Conus ritae* Petuch, 1995, which is only known from a few specimens [8]. Besides having an overall shape that is similar to *C*. *ritae*, the coloration patterning of both species closely match (see [8], pl. 4). Specimens of *C*. *franklinae*, however, have narrower last whorls than specimens of *C*. *ritae* (Kohn [8] reported RD values for *C*. *ritae* that range from 0.61–0.68). Puillandre et al. [2] assigned *C*. *ritae* to the subgenus *Atlanticonus*, along with three other species: *C*. *cuna* Petuch, 1998, *C*. *granulatus* Linnaeus, 1758, and *C*. *glenni* Petuch, 1993 (none of these four extant species of *Atlanticonus* have yet been placed in an explicit phylogenetic context). This subgeneric assignment is tentatively also followed here for *C*. *franklinae*.

**Subgenus *Stephanoconus* Mörch, 1852**

Type species: *Conus regius* Gmelin, 1791.

***Conus* (*Stephanoconus*) *gouldi* Hendricks sp. nov**.

urn:lsid:zoobank.org:act:0436FD65-9526-44F9-8FA3-E2AAA537101A

**Fig 14:**
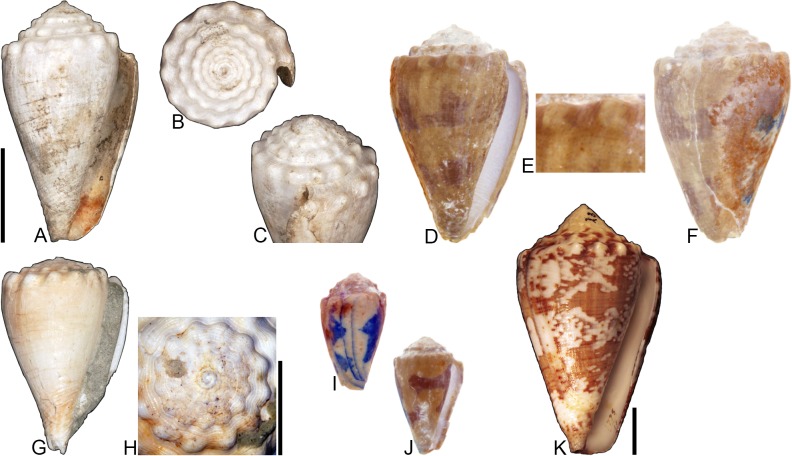
*Conus* (*Stephanoconus*) *gouldi* Hendricks sp. nov. (A-J) and *Conus* (*Stephanoconus*) *regius* Gmelin, 1791 (K). Fossil specimens are from locality station TU 1422 (Cercado Fm.); modern *C*. *regius* is from the West Indies. (A-C) PRI 66168 (holotype), SL 24.3 mm; (D-F) PRI 66193 (paratype), SL 22.3 mm; (G-H) PRI 66194 (paratype), SL 19.8 mm; (I-J) PRI 66197 (paratype), SL 12.0 mm; (K) CASIZ 178046, 53.1 mm. (D-F, I-J) are reversed images of specimens photographed under UV light. Scale bar to the left of (A) is 1 cm and pertains to (A-D, F-G, I-J); (E) is a magnified view of the shoulder region of (D), illustrating pigmentation between tubercles; (H) scale bar is 5 mm; (K) scale bar is 1 cm and pertains to that image.

Fig. 14A-J, S2 Table

### Material examined

**Holotype**: PRI 66168. **Paratypes**: PRI 66193–66197. All type specimens are from TU station 1422.

### Type locality and horizon

TU 1422: Arroyo Bellaco, Dominican Republic; upper Miocene Cercado Formation.

### Description

#### Shell size

Shell moderately small (largest observed specimen, PRI 66196, is 26.7 mm; this specimen is missing a small portion of its spire and anterior end).

#### Last whorl

Broadly and ventricosely conical or broadly conical (RD 0.70–0.75, μ = 0.72; PMD 0.81–0.88, μ = 0.84; n = 5); outline convex to sigmoidal. Shoulder subangulate, tuberculate. Most shells widest below shoulder. Aperture uniform in width from base to shoulder. Siphonal notch absent. Fine spiral threads restricted to the basal half; some specimens exhibit a fine spiral groove that cuts across shoulder tubercles.

#### Spire whorls

Spire height low to moderate (RSH 0.10–0.23, μ = 0.18; n = 5); outline straight to slightly convex. Protoconch unknown. All postnuclear whorls tuberculate. Sutural ramp concave to sigmoidal, with multiple spiral threads. Subsutural flexure asymmetrical, depth about 1.7x width.

#### Coloration pattern

Description based on mature specimen PRI 66193 (Fig. 14D-F). Two noninteracting patterns present. The primary (base) pattern consists of two irregular spiral bands on the last whorl; occasional axial blotches extend toward the shoulder from the posterior band. Regions between the shoulder tubercles are also pigmented. The secondary pattern consists of many (>30; poor preservation prevents an accurate count) spiral rows of fine dots that extend from the base to just below the shoulder; at the shoulder, these appear to coalesce into fine axial streaks. The two patterns differ slightly in the color of emitted light. Pattern on sutural ramp unknown. Immature specimen PRI 66197 (Fig. 14I,J) has irregular blotches on the last whorl and lacks the secondary pattern.

### Etymology

Named for Stephen J. Gould (1941–2002) in recognition of his important contributions to paleontology.

### Remarks

*Conus gouldi* is unlike co-occurring fossil species, but is very similar in shell morphology to the extant western Atlantic species *Conus regius* (Fig. 14K), as recently circumscribed by Kohn [8]. Coloration patterns are not well preserved in the known specimens of *C*. *gouldi*, but PRI 66193 does show last whorl patterning similar to some adult specimens of *C*. *regius* (e.g., [8], pl. 40, Figs. 8, 9). Furthermore, juvenile specimens of *C*. *regius* (e.g., [8], pl. 42, Figs. 9–11) sometimes lack the spiral rows of dots that are present in mature specimens, something also observed here in *C*. *gouldi* (PRI 66197). An important difference between the fossil and extant species, however, is adult shell size; *C*. *regius* reaches a “[t]ypical” shell length of 43 mm ([8], p. 179; the largest known specimen is over 80 mm), but the largest specimen of *C*. *gouldi* is only 26.7 mm (it is missing a small portion of its spire and anterior end, however).

Puillandre et al. [1] demonstrated a close relationship between *C*. *regius* and the sister taxa *C*. *bartschi* Hanna and Strong, 1949 and *C*. *brunneus* Wood, 1828, all three of which they assigned to the subgenus *Stephanoconus* Mörch, 1852 (along with seven additional species). This subgeneric assignment is followed here for *C*. *gouldi*.

***Conus* (*Stephanoconus*) *sewalli* Maury, 1917** [40]

Fig. 15A-I

**Fig 15:**
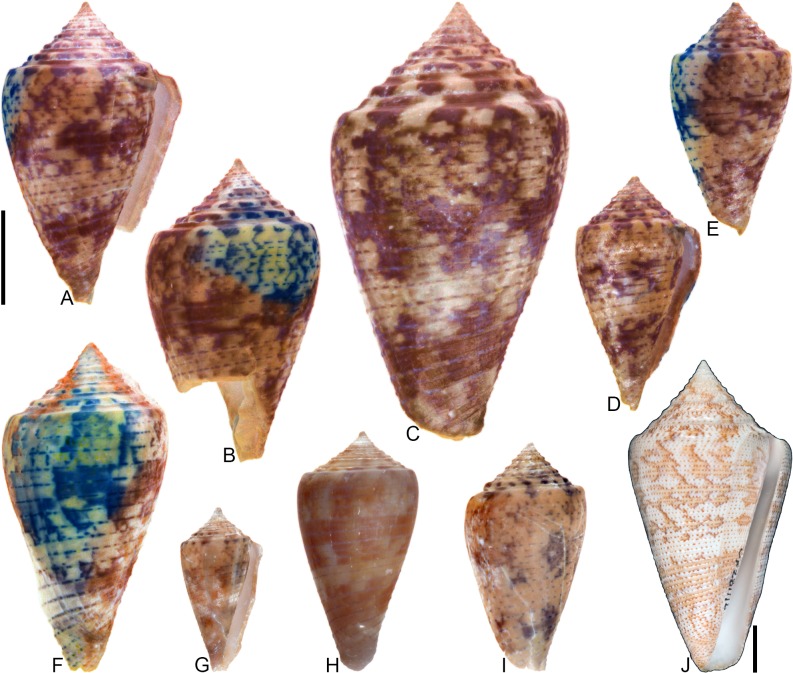
*Conus* (*Stephanoconus*) *sewalli* (Maury, 1917) (A-I) and *Conus* (*Stephanoconus*) *mappa* [Lightfoot, 1786] (J). Fossil specimens are from locality stations TU 1422 (Cercado Fm.) and TU 1215 (Gurabo Fm.); modern *C*. *mappa* is from Pigeon Point, Tobago. (A-B) PRI 67183, TU 1422, SL 30.6 mm; (C) PRI 66160, TU 1422, SL 44.1 mm; (D-E) PRI 67193, TU 1422, SL 23.5 mm; (F) PRI 67184, TU 1422, SL 33.3 mm; (G) PRI 67565, TU 1215, SL 17.3 mm; (H) PRI 67564, TU 1215, SL 25.2 mm; (I) PRI 66152, TU 1215, SL 24.1 mm; (J) UF 281116–1, SL 64.4 mm. (A-I) are reversed images photographed under UV light. Scale bar to left of (A) is 1 cm and pertains to (A-I). Scale bar to right of (J) is 1 cm and pertains to that image.

*Conus sewalli* Maury, 1917 [40], p. 201–202, pl. 5, Fig. 3, pl. 6, Fig. 3.

*Purpuriconus sewalli* (Maury), Tucker and Tenorio, 2009 [34], p. 116.

### Material examined

**Syntype**: PRI 28599. **Other specimens**: PRI 66160, PRI 66163, PRI 67183, PRI 67184, PRI 67193 (TU station 1422); PRI 66152, PRI 67564–67565 (TU 1215); PRI 66182 (TU 1354).

### Coloration pattern

Two weakly interacting patterns present. The primary (base) pattern consists of two elements: 1) both large and small irregularly shaped blotches concentrated into two regions on the last whorl—near the anterior end of the shell and below the PMD of the last whorl, leaving an unpigmented region just below the center; 2) small sub-triangular markings concentrated on the posterior third of the last whorl. The secondary pattern consists of 17–23 spiral rows of dots and dashes extending from the base to shoulder; spaces between dashes are sometimes unpigmented in instances where the secondary pattern overlies the primary pattern. The secondary pattern coincides with spiral ornamentation features on the anterior end of the shell. The two patterns differ in the color of emitted light. The axial blotches associated with the primary pattern sometimes extend over the shoulder onto the sutural ramp.

### Remarks

*Conus sewalli* is similar in shell form to *C*. *haytensis* Sowerby I, 1850 (see below), another Dominican Neogene species, and the two taxa can be challenging to differentiate without observation of their coloration patterns. Most significantly, *C*. *sewalli* has sub-triangular markings on the last whorl, but these are absent in *C*. *haytensis*. Furthermore, the last whorl shape of *C*. *sewalli* tends to be more convex than that of *C*. *haytensis*, which is often slightly sigmoidal in profile. The coloration pattern of *C*. *sewalli* is similar to *C*. *bellacoensis* sp. nov. (see below), as well as extant species such as *C*. *mappa* (Figs. 1, 15J) and *C*. *curassaviensis* (see [8], pl. 39, Fig. 18) that are members of the western Atlantic “*Conus cedonulli* Species Group” (see [8], p. 144). For additional discussion of this association, see remarks below for *C*. *bellacoensis* sp. nov. Tucker and Tenorio [34] placed *C*. *sewalli* in the genus *Purpuriconus* da Motta, 1991, but the strong similarity between *C*. *sewalli* and extant species like *C*. *mappa* instead suggest that it belongs in the subgenus *Stephanoconus* (see additional remarks below for *C*. *bellacoensis* sp. nov.).

***Conus* (*Stephanoconus*) *bellacoensis* Hendricks sp. nov**.

urn:lsid:zoobank.org:act:DEAA465A-EED2-4DE4-9185-6D7DD41F8C7F

**Fig 16:**
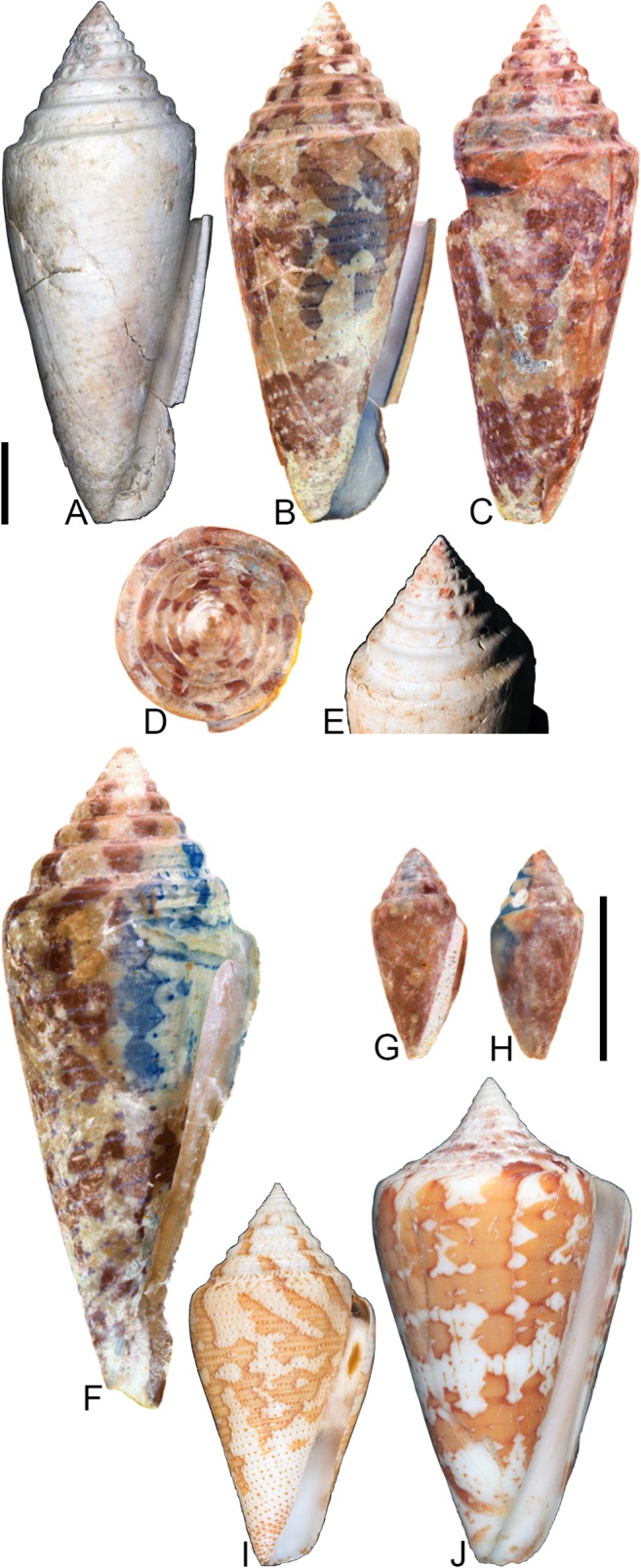
*Conus* (*Stephanoconus*) *bellacoensis* Hendricks sp. nov. (A-H), *Conus* (*Stephanoconus*) *mappa* [Lightfoot, 1786] (I), and *Conus* (*Stephanoconus*) *archon* Broderip, 1833 (J). Fossil specimens are from locality stations TU 1422 (Cercado Fm.) and TU 1354 (Gurabo Fm.); modern *C*. *mappa is* from Pigeon Point, Tobago; modern *C*. *archon* is from the Gulf of California, Mexico. (A-E) PRI 66158 (holotype), TU 1422, SL 59.7 mm; (F) PRI 67252 (paratype), TU 1354, SL 76.0 mm; (G-H) PRI 67186 (paratype), TU 1422, SL 12.4 mm; (I) UF 241414, SL 45.5 mm; (J) UF 26040, SL 58.7 mm. (B-D, F-H) are reversed images photographed under UV light. Scale bar to left of (A) is 1 cm and pertains to (A-F, I-J). Scale bar to right of (H) is 1 cm and pertains to (G-H).

Fig. 16A-H, S2 Table

### Material examined

**Holotype**: PRI 66158 (TU station 1422). **Paratypes**: PRI 67186 (TU station 1422); PRI 67252 (TU station 1354).

### Type locality and horizon

TU 1422: Arroyo Bellaco, Dominican Republic; upper Miocene Cercado Formation.

### Other locality and horizon

TU 1354: Cañada de Zamba, Dominican Republic; lower Pliocene Gurabo Formation.

### Description

#### Shell size

Shell moderately large (largest observed specimen, PRI 67252, is 76.0 mm).

#### Last whorl

Conical (RD 0.53–0.66, μ = 0.57; PMD 0.88–0.93, μ = 0.91; n = 3); outline slightly convex. Shoulder angulate; smooth in mature specimens. Widest part of shell at shoulder in juvenile specimens, below shoulder in mature specimens. Aperture slightly wider at base than near shoulder. Siphonal notch absent. Weakly beaded spiral threads on anterior half.

#### Spire whorls

Spire height high (RSH 0.25–0.31, μ = 0.28; n = 3); outline straight, whorls stepped. Protoconch unknown. Most spire whorls tuberculate (information unavailable on earliest post nuclear whorls; 5 tuberculate whorls preserved on PRI 67252, 7 on PRI 66158), though later whorls are not. Sutural ramp concave, unornamented. Subsutural flexure symmetrical, depth about 3x width.

#### Coloration pattern

Two interacting patterns (one complex, one simple) present; it is not clear which pattern is primary. The more complex pattern consists of an irregular spiral band on the anterior end of the last whorl and a complex posterior pigmentation region composed of diamond-shaped blotches that are linked along their edges or corners to form axial or oblique-axial streaks. The simpler pattern consists of about 20 rows of spiral dots; spaces between dots sometimes appear to be unpigmented. The spiral dots are more closely spaced in regions where they intersect the complex pattern. The two patterns differ in the color of emitted light. Axial streaks on the last whorl sometimes extend onto the sutural ramp.

### Etymology

Named for the type locality, Arroyo Bellaco.

### Remarks

*Conus bellacoensis* is similar in shell form—and especially in its complex coloration pattern—to members of the “*Conus cedonulli* Species Group” ([8], p. 144), which includes the western Atlantic species *C*. *cedonulli* Linnaeus, 1767, *C*. *pseudaurantius* Vink and von Cosel, 1985, *C*. *aurantius* Hwass in Bruguière, 1792, *C*. *mappa* [Lightfoot, 1786] (Figs. 1F, 15J, 16I), and *C*. *curassaviensis* Bruguière, 1792 [8]. The phylogenetic analysis of Puillandre et al. [1] also recognized the eastern Pacific species *C*. *archon* (Figs. 1E, 16J) as closely related to three of these species: (*C*. *archon*(*C*. *cedunolli*(*C*. *curassaviensis*,*C*. *mappa*))). Puillandre et al. [2] assigned these extant species to the subgenus *Stephanoconus*, a designation that is followed here for *C*. *bellacoensis*, based on the hypothesis that it is also a member of this group. A key difference that separates the new species from extant members of this group is its narrower last whorl: the two known adult shells (PRI 66158 and PRI 67252) have relative diameters of 0.53, while other species in the group all have wider last whorls (mean values range from 0.60 to 0.66; [8]). Among Neogene fossils from the Dominican Republic, *C*. *bellacoensis* is most similar to *C*. *sewalli* (Fig. 15A-I), a species that is also hypothesized to be a member of the *Stephanoconus* clade. *Conus bellacoensis* can be differentiated from *C*. *sewalli* by its narrower last whorl and higher spire.

**Subgenus *Pyruconus* Olsson, 1967**

Type species: *Conus patricius* Hinds, 1843.

***Conus* (*Pyruconus*) *recognitus* Guppy, 1867** [47]

Fig. 17A-D

**Fig 17:**
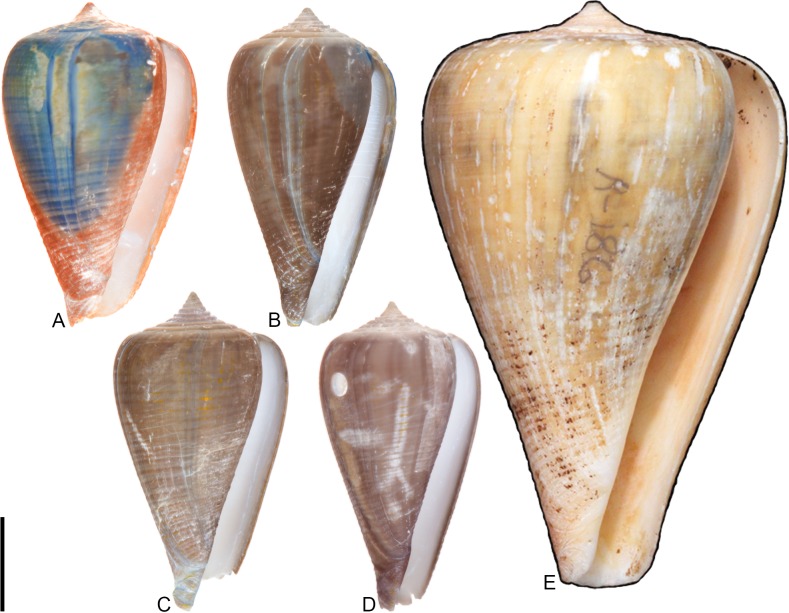
*Conus* (*Pyruconus*) *recognitus* Guppy, 1866 (A-D) and *Conus* (*Pyruconus*) *patricius* Hinds, 1843 (E). Fossil specimens are from locality station TU 1354 (Gurabo Fm.); modern *C*. *patricius* is from Palo Seco, Panama. (A) PRI 67539, SL 33.8 mm; (B) PRI 67538, SL 33.4 mm; (C) PRI 67537, SL 33.4 mm; (D) PRI 66181, SL 32.4 mm; (E) UF 321278–1, SL 60.8 mm. (A-D) are reversed images of specimens photographed under UV light. Scale bar is 1 cm and pertains to all images.

*Conus solidus* of Sowerby I, 1850 [37], p. 45; Guppy, 1866 [38], p. 287, pl. 16, Fig. 1; not Gmelin, 1791, not Sowerby I, 1841.

*Conus recognitus* Guppy, 1867 [47], p. 171; Maury, 1917 [40], p. 209, pl. 7, Fig. 9; Pilsbry, 1921 [39], pl. 19, Fig. 2.

*Conus* (*Lithoconus*) *recognitus* Guppy, Pflug, 1961 [45], pl. 18, Figs. 12–15.

*Pyruconus recognitus* (Guppy), Tucker and Tenorio, 2009 [34], p. 117.

### Material examined

**Syntype**: NHMUK PI BM G 83970. **Other specimens**: PRI 66181, PRI 67537–67541 (TU station 1354).

### Coloration pattern

One pattern present. Pattern consists of a solid, undifferentiated ground pattern that extends over the entire last whorl. The last whorl pattern extends over the shoulder onto the sutural ramp.

### Remarks

The specimens of *C*. *recognitus* illustrated here (Fig. 17A-D) do fluoresce, but do not show evidence of a distinctive pattern, supporting the description provided above of a solidly pigmented ground pattern. The distinctive shell shape of *C*. *recognitus* separates the species from co-occurring fossil species. *Conus recognitus* is very similar in shell form to the extant eastern Pacific species *C*. *patricius* Hinds, 1843, an association also recognized by Woodring [48]. The coloration pattern of *Conus patricius* (e.g., Fig. 17E) consists of a solid, cream-colored base pattern that sometimes also exhibits subtle spiral and/or axial bands of slightly different color. The patterning of *C*. *patricius* explains the undifferentiated regions of fluorescence shown here in *C*. *recognitus* (Fig. 17A-D).

Tucker and Tenorio [34] assigned *C*. *recognitus* to the genus *Pyruconus* Olsson, 1967, which Puillandre et al. [2] recognized as a subgeneric ranking. This assignment is followed here for *C*. *recognitus* (see remarks for *C*. *haytensis*, which discuss the paraphyletic nature of *Pyruconus* as a genus-level name when applied to the extant species *C*. *fergusoni* Sowerby III, 1873).

***Conus* (“*Pyruconus*”) *haytensis* Sowerby I, 1850** [37]

Fig. 18

**Fig 18:**
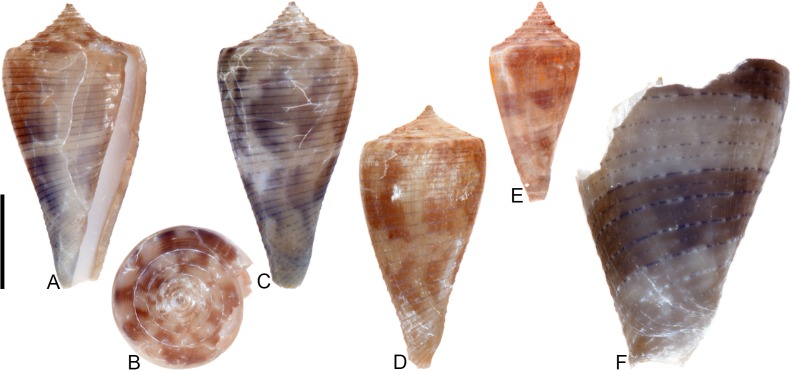
*Conus* (“*Pyruconus*”) *haytensis* Sowerby I, 1850. All specimens are from locality station TU 1215 (Gurabo Fm.). (A-C) PRI 67552, SL 30.2 mm; (D) PRI 67551, SL 27.9 mm; (E) PRI 67554, SL 21.2 mm; (F) *Conus haytensis*?, PRI 67555, fragment is 33.6 mm in length. All are reversed images of specimens photographed under UV light. Scale bar is 1 cm and pertains to all images.

*Conus haytensis* Sowerby I, 1850 [37], p. 44; Pilsbry, 1921 [39], p. 326, pl. 19, Fig. 1.

*Conus* (*Dendroconus*) *haytensis* Sowerby I, Pflug, 1961 [45], pl. 16, Figs. 1–5.

### Material examined

**Lectotype**: NHMUK PI BM G83961. **Other specimens**: PRI 66139, PRI 67551–67554, questionably PRI 67555 (TU station 1215).

### Coloration pattern

Two noninteracting patterns present. The primary (base) pattern consists of 2–3 discontinuous (forming broad axial streaks) or continuous spiral bands. The secondary pattern consists of 14–27 (number increases with shell size) spiral rows of lines. The two patterns differ in the color of emitted light. Sutural ramp with radial blotches that sometimes appear to be extensions of the primary pattern over the shoulder.

### Remarks

In terms of shell characters, *C*. *haytensis* may be easily confused with the fossil species *C*. *sewalli*, but these two species have very different coloration patterns (compare Figs. 15 and 18). Tucker and Tenorio [34] assigned *C*. *haytensis* to the genus *Pyruconus* Olsson, 1967. Puillandre et al. [1], however, showed that “*Pyruconus*” is paraphyletic (also see [2]), as the two extant species—*C*. *patricius* Hinds, 1843 and *C*. *fergusoni* Sowerby III, 1873, both of which are from the eastern Pacific—assigned to this genus-level grouping by Tucker and Tenorio [34] nest in different clades. Nevertheless, Puillandre et al. [2] assigned both species to *Pyruconus* (ranked at the subgeneric-level) “to avoid the creation of a new subgeneric name” (p. 12). *Conus haytensis* is more similar to *C*. *fergusoni* (e.g., http://www.coneshell.net/pages/c_fergusoni.htm) than to *C*. *patricius* (see the above description of *C*. *recognitus*, a species similar to *C*. *patricius*, as well as the photograph of *C*. *patricius* in Fig. 17A-D). As *C*. *patricius* is the type species of *Pyruconus* [2], *C*. *fergusoni* and *C*. *haytensis* should probably be assigned to a different subgenus of cone snails; nevertheless, the paraphyletic subgenus name “*Pyruconus*” is used here for consistency with Tucker and Tenorio [34] and Puillandre et al. [2] until this taxonomic matter can be formally resolved.

Specimen PRI 67555 (Fig. 18F) may be a fragment of a much larger specimen of *C*. *haytensis* than the other specimens shown here (*C*. *haytensis* is one of the largest species of fossil cone snails known from the Dominican Republic). If so, its secondary coloration pattern shows variation not exhibited by the other, smaller specimens: rows of spiral dots and dashes, with unpigmented regions in-between.

**Subgenus *Ductoconus* da Motta, 1991**

Type species: *Conus princeps* Linnaeus, 1758.

***Conus* (*Ductoconus*) *cashi* Hendricks sp. nov**.

urn:lsid:zoobank.org:act:4E5E81F2-9F92-4BD7-AA62-200499FCB5AE

**Fig 19:**
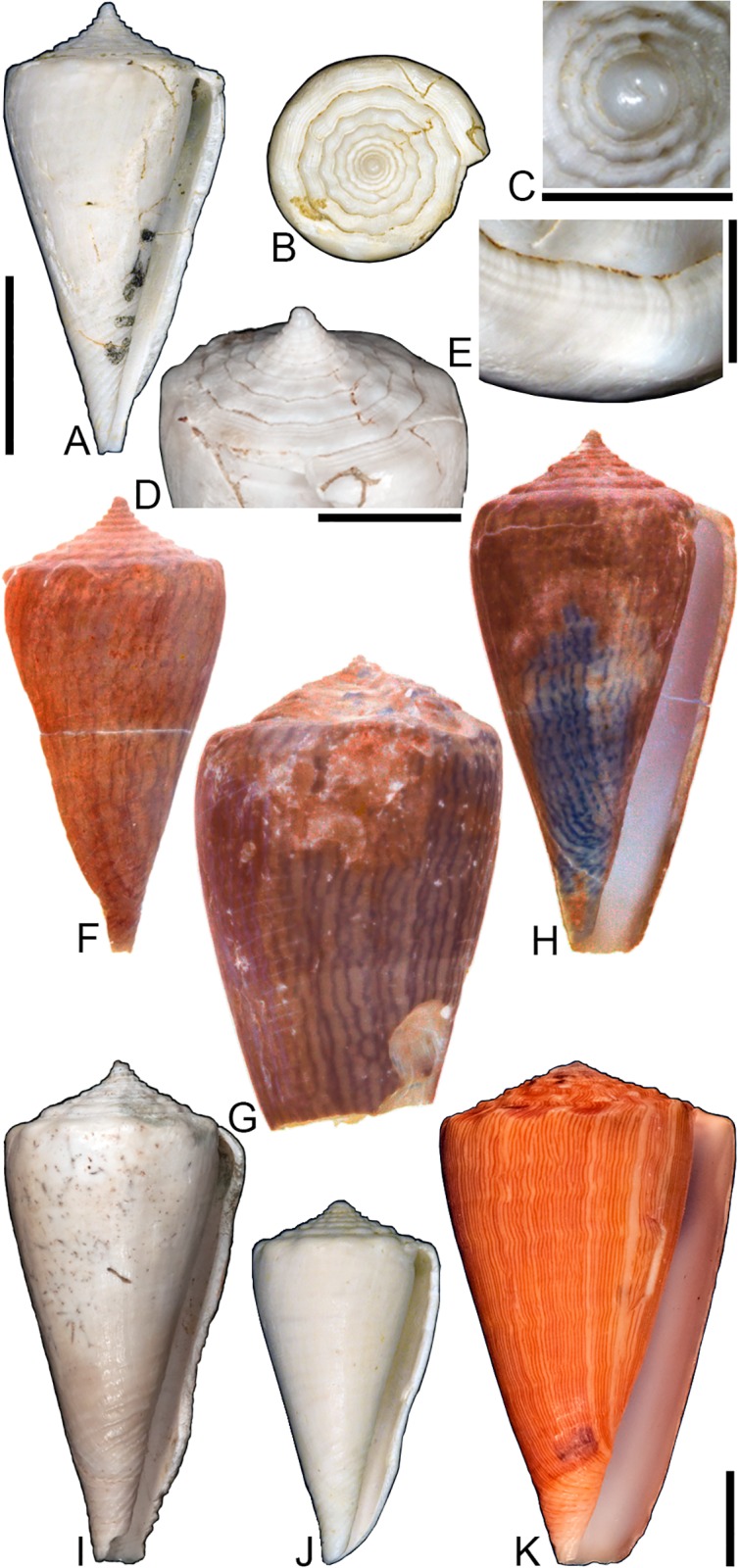
*Conus* (*Ductoconus*) *cashi* Hendricks sp. nov. (A-J) and *Conus princeps* Linnaeus, 1758 (K). Fossil specimens are from locality stations TU 1422 (Cercado Fm.), TU 1215 (Gurabo Fm.), TU 1278 (Gurabo Fm.), and NMB 15848 (Gurabo Fm.); modern *C*. *princeps* is from the Perlas Islands, Panama. (A-F) PRI 66144, TU 1215, SL 22.5 mm; (G) PRI 67166, TU 1422, SL 23.8 mm; (H) PRI 66162, TU 1422, SL 26.2 mm; (I) NMB H18381, NMB 15848, SL 25.0 mm; (J) PRI 66116, TU 1278, SL 18.1 mm; (K) UF 169052–1, SL 52.2 mm. (F-H) are reversed images photographed under UV light. Scale bar to the left of (A) is 1 cm and pertains to (A-B, F-J); (C, E) scale bars are 2 mm; (D) scale bar is 5 mm; (K) scale bar is 1 cm.

Fig. 19A-J, S2 Table

### Material examined

**Holotype**: PRI 66144 (TU station 1215). **Paratypes**: PRI 66162 and PRI 67166 (TU station 1422); PRI 66116 (TU station 1278); NMB H18381 (NMB locality 15848).

### Type locality and horizon

TU 1215: Río Gurabo, Dominican Republic; lower Pliocene Gurabo Formation.

### Other localities and horizons

TU 1422: Arroyo Bellaco, Dominican Republic; upper Miocene Cercado Formation. TU1278: “Large arroyo on east side of Río Gurabo just at the ford on Los Quemados-Sabaneta road” (Saunders et al. [24], p. 65); lower Pliocene Gurabo Formation. NMB 15848: Río Gurabo, lower Pliocene Gurabo Formation (see text-Fig. 4 in [24]); locality represents a subset of TU 1215.

### Description

#### Shell size

Shell moderately small (largest complete observed specimen, PRI 66162, is 26.2 mm; PRI 67166, which is missing its anterior third, is larger).

#### Last whorl

Narrowly conical to conical (RD 0.49–0.50, μ = 0.49; PMD 0.85–0.86, μ = 0.86; n = 2); outline convex on posterior half, slightly concave on anterior half, resulting in slightly sigmoidal profile. Shoulder angulate to subangulate; tuberculate. Widest part of shell below shoulder. Aperture uniform in width from base to shoulder. Siphonal notch absent. Spiral threads on anterior third.

#### Spire whorls

Spire height moderate (RSH 0.14; n = 2); outline concave to sigmoidal. Protoconch with ca. 2 whorls, diameter 0.9 mm (based on PRI 66144). All postnuclear whorls tuberculate. Sutural ramp sigmoidal, with 2–3 spiral threads. Subsutural flexure slightly asymmetrical, depth about 0.5x width.

#### Coloration pattern

Two noninteracting patterns present. The primary (base) pattern consists of two wide spiral bands that cover most of the anterior and posterior ends of the last whorl, leaving a narrow, unpigmented band in the center. The secondary pattern consists of jagged, non-branching (but sometimes touching) thin axial streaks that in many cases extend from the base to the shoulder. The two patterns differ slightly in the color of emitted light. Sutural ramp with radial streaks that roughly correspond with the shape of the subsutural flexure.

### Etymology

Named in honor of American musician John “Johnny” Cash (1932–2003), otherwise known as “The Man in Black.”

### Remarks

*Conus cashi* is not similar to other DR fossil species, but is similar in shell morphology and especially coloration pattern to the extant eastern Pacific species *Conus* (*Ductoconus*) *princeps* Linnaeus, 1758 (Fig. 19K), suggesting that *C*. *cashi* should also be included with *C*. *princeps* in the subgenus *Ductoconus* da Motta, 1991, which was accepted by Puillandre et al. [2]. Puillandre et al. [1] found molecular support for the western Atlantic species *C*. *hieroglyphus* Duclos, 1833 being the sister taxon of *C*. *princeps*, but it has a very different shell morphology than *C*. *princeps*.

**Subgenus *Dauciconus* Cotton, 1945**

Type species: *Conus daucus* Hwass, 1792.

***Conus* (*Dauciconus*) *furvoides* Gabb, 1873** [49]

Fig. 20

**Fig 20:**
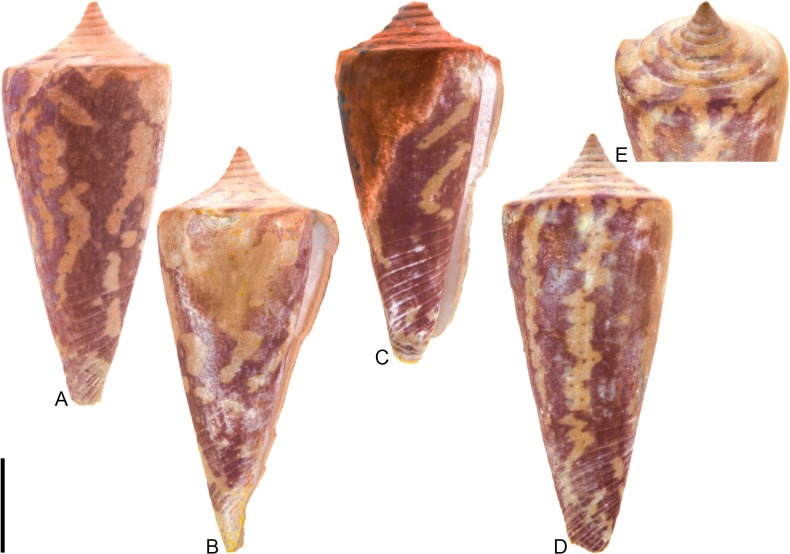
*Conus* (*Dauciconus*) *furvoides* Gabb, 1873. All specimens are from locality station TU 1354 (Gurabo Fm.). (A-B) PRI 67239, SL 40.8 mm; (C) PRI 67238, SL 36.6 mm; (D-E) PRI 67240, SL 42.3 mm. All are reversed images photographed under UV light. Scale bar is 1 cm and pertains to all images.

*Conus furvoides* Gabb, 1873 [49], p. 232; Maury, 1917 [40], p. 206–207, pl. 7, Figs. 1, 2; Pilsbry, 1921 [39], p. 328, pl. 20, Fig. 1.

*Dauciconus furvoides* (Gabb), Tucker and Tenorio, 2009 [34], p. 89.

### Material examined

**Lectotype**: ANSP 2576. **Other specimens**: PRI 66188, PRI 67238–67241 (TU station 1354).

### Coloration pattern

Two weakly interacting patterns (one complex, one simple) present; it is not clear which pattern is primary. The more complex pattern consists of axially arranged markings that extend from the base to shoulder and onto the sutural ramp. These markings may also cover broader areas of the last whorl, forming unpigmented axial or diagonal blotches. PRI 67240 (Fig. 20D) shows evidence of the temporary cessation of the complex pigmentation field (though not necessarily cessation of shell production) in the direction of growth, followed by its later resumption, resulting in oppositely-patterned regions that appear like puzzle pieces that might fit together. The simpler pattern consists of at least 12–17 spiral rows of dots (the extent of this pattern on the anterior third of the last whorl is not known). Interaction between the two coloration patterns occurs when the spiral dots intersect the margins of the axial patterns; this results in the formation of small, triangular shaped blotches. The two patterns do not differ in the color of emitted light. The complex last whorl pattern extends over the shoulder onto the sutural ramp.

### Remarks

*Conus furvoides* has cannot be easily confused with co-occurring fossil species. It does, however, shares similarities in shell form with some extant members of the subgenus *Dauciconus* Cotton, 1945 (sensu [2]) (Tucker and Tenorio [34] assigned *C*. *furvoides* to *Dauciconus*, although at the genus level). Some specimens of *C*. (*Dauciconus*) *virgatus* Reeve, 1849, an eastern Pacific species, exhibit the puzzle-piece like arrangement of axial streaks described above (e.g., http://www.gastropods.com/9/Shell_769.shtml), while some specimens of *C*. (*Dauciconus*) *recurvus* Broderip and Sowerby, 1833—an eastern Pacific species that is the sister taxon of *C*. *virgatus* [1]—show evidence of diagonal blotches (e.g., http://www.coneshell.net/pages/c_recurvus.htm). Neither *C*. *virgatus*, nor *C*. *recurvus*, however, show evidence of rows of spiral dots on the last whorl, which are observed in *C*. *furvoides*. *Conus furvoides* is also similar in shell shape to *C*. *villepinii* Fischer and Bernardi, 1857, an extant western Atlantic species also assigned by Puillandre et al. [2] to the subgenus *Dauciconus*. The coloration pattern of *C*. *furvoides*, however, differs substantially from the specimens of *C*. *villepinii* figured by Kohn [8]; in particular, the last whorl coloration pattern of *C*. *villepinii*—which consists of two discontinuous spiral bands, resulting in axial streaks—is not present in *C*. *furvoides*.

***Conus* (*Dauciconus*) *planiliratus* Sowerby I, 1850** [37]

Fig. 21

**Fig 21:**
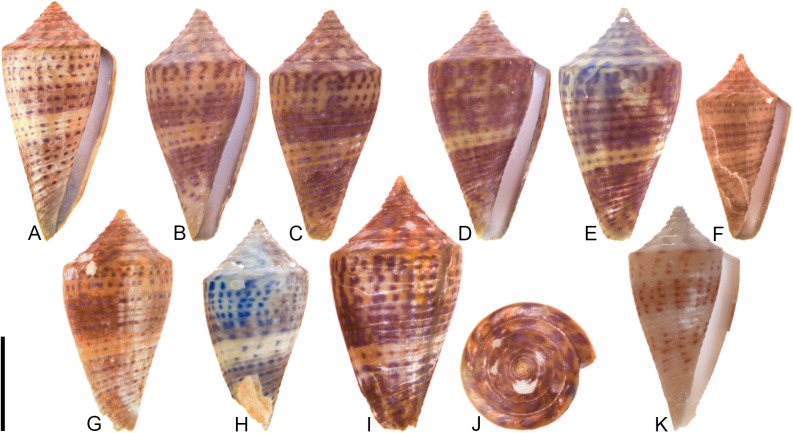
*Conus* (*Dauciconus*) *planiliratus* Sowerby I, 1850. Specimens are from locality stations TU 1215 and TU 1354 (both Gurabo Fm.). (A) PRI 67502, TU 1354, SL 24.0 mm; (B-C) PRI 66179, TU 1354, SL 23.4 mm; (D-E) PRI 67504, TU 1354, SL 23.2 mm; (F) PRI 67508, TU 1354, SL 18.9 mm; (G) PRI 67505, TU 1354, SL 22.4 mm; (H) PRI 67503, TU 1354, SL 21.3 mm; (I-J) PRI 67509, TU 1354, SL 25.8 mm; (K) PRI 66146, TU 1215, SL 23.9 mm. All are reversed images photographed under UV light. Scale bar is 1 cm and pertains to all images.

*Conus planiliratus* Sowerby I, 1850 [37], p. 44; Guppy, 1866 [38], p. 287, pl. 16, Fig. 7; Pilsbry, 1921 [39], p. 329–330, pl. 20, Figs. 6, 9.

*Conasprelloides planiliratus* (Sowerby I), Tucker and Tenorio, 2009 [34], p. 88.

### Material examined

**Syntypes**: NHMUK PI BM GG 20131–20134. **Other specimens**: PRI 66146, PRI 66151, and PRI 67548 (TU station 1215); PRI 66176, PRI 66179, PRI 67502–67509, questionably PRI 67506 (TU station 1354).

### Coloration pattern

Two noninteracting patterns present. The primary (base) pattern consists of two solid bands: one on the anterior half of the last whorl, the other on the posterior half, but below the shoulder. The secondary pattern consists of about 17–20 spiral rows of dots (which are often square-shaped) or dashes; these sometimes coalesce just below the shoulder to form short axial streaks. The two patterns differ in the color of emitted light. Sutural ramp with radial blotches; some of these appear to be extensions of the secondary pattern (axial streaks) over the shoulder.

### Remarks

*Conus planiliratus* can be distinguished from co-occurring fossil species by the presence of spiral ribs over much of its last whorl, the presence of spiral threads on its sutural ramp, and its distinctive coloration pattern. Among extant western Atlantic species, *C*. *planiliratus* bears strong similarity in shell morphology to *C*. *cancellatus* Hwass in Bruguière, 1792 and *C*. *stimpsoni* Dall, 1902, both of which were recently characterized and compared by Kohn [8]. *Conus planiliratus* differs from both of these species in its coloration pattern, which features a heavily pigmented secondary pattern (spiral rows of dots and dashes); this is not present in either of the extant species. Tucker and Tenorio [34] placed *C*. *planiliratus* in the genus *Conasprelloides* Tucker and Tenorio, 2009, which Puillandre et al. [2] considered synonymous with the subgenus *Dauciconus*. Puillandre et al. [2] placed both *C*. *cancellatus* and *C*. *stimpsoni* in the subgenus *Dauciconus* and this assignment is followed here for *C*. *planiliratus*.

***Conus* (*Dauciconus*) *multiliratus* Böse, 1906** [50]

Fig. 22

**Fig 22:**
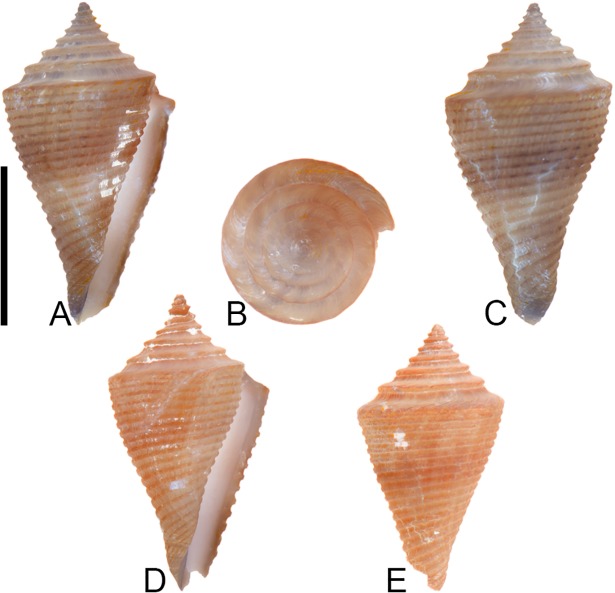
*Conus* (*Dauciconus*) *multiliratus* Böse, 1906. All specimens are from locality station TU 1215 (Gurabo Fm.). (A-C) PRI 67572, SL 20.2 mm; (D) PRI 66147, SL 18.5 mm; (E) PRI 67577, SL 16.7 mm. All are reversed image of specimens photographed under UV light. Scale bar is 1 cm and pertains to all images.

*Conus agassizi multiliratus* Böse, 1906 [50], p. 49–50, pl. 5, Figs. 34–38.

*Conus* (*Leptoconus*) *multiliratus* Marks, 1951 [51], p. 139.

*Conus gaza* Johnson and Pilsbry, 1911 [52], p. 342–343, pl. 23, Figs. 2, 3; Maury, 1917 [40], p. 210–211, pl. 7, Fig. 12.

*Conasprelloides multiliratus* (Böse), Tucker and Tenorio, 2009 [34], p. 88.

### Material examined

PRI 66147, PRI 67572–67577 (TU station 1215); PRI 66174 (TU station 1354).

### Coloration pattern

Probably two noninteracting patterns present. The primary (base) pattern consists of two continuous spiral bands, one at the base of the shell and one near the midpoint of the last whorl; these are lightly pigmented. The secondary pattern (which requires confirmation in specimens with better preserved patterns) seems to consist of spiral rows of dashes associated with the ribs that cover the last whorl. The two patterns differ slightly in the color of emitted light. Sutural ramp with lightly pigmented radial blotches.

### Remarks

The distinctive, heavily-ribbed shell of *Conus multiliratus* cannot be confused with any co-occurring species. Tucker and Tenorio [34] assigned *C*. *multiliratus* to the genus *Conasprelloides*, which Puillandre et al. [2] recognized as a synonym of the subgenus *Dauciconus*. *Conus multiliratus* is similar in shell shape to the extant western Atlantic species *C*. *cancellatus*, which Puillandre et al. [2] assigned to *Dauciconus* based on phylogenetic evidence [1]. Additionally, some specimens of *C*. *cancellatus* (e.g., [8], pl. 70, Figs. 1–4) have two lightly pigmented spiral bands on the last whorl that are similar to those shown here in *C*. *multiliratus*. An important difference between *C*. *multiliratus* and *C*. *cancellatus*, however, is that the extinct species lacks spiral ornamentation on the sutural ramp, while it is present in *C*. *cancellatus* [8].

***Conus* (*Dauciconus*) *karlschmidti*? Maury, 1917** [40]

Fig. 23

**Fig 23:**
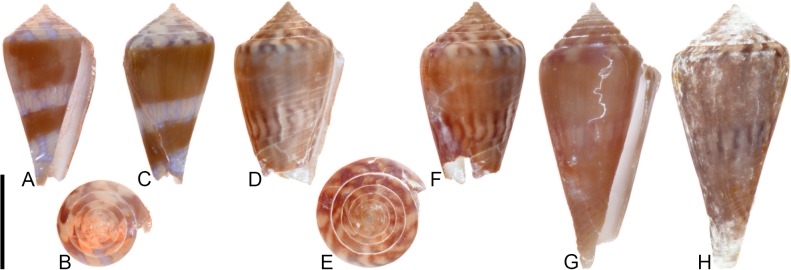
*Conus* (*Dauciconus*) *karlschmidti*? Maury, 1917. All specimens are from locality station TU 1215 (Gurabo Fm.). (A-C) PRI 67557, SL 19.1 mm; (D-F) PRI 67562, SL 19.5 mm (shell broken); (G) PRI 66138, SL 27.9 mm; (H) PRI 67561, SL 27.9 mm. All are reversed images photographed under UV light. Scale bar is 1 cm and pertains to all images.

*Conus karlschmidti* Maury, 1917 [40], p. 211–212, pl. 7, Fig. 14.

### Material examined

PRI 66138, PRI 67556–67563 (TU station 1215).

### Coloration pattern

Two noninteracting patterns present. The primary (base) pattern consists of wavy, sometimes branching axial streaks that extend from the base to shoulder. The secondary pattern consists of two spiral bands that cover the anterior and posterior thirds of the last whorl, exposing the primary pattern in-between; in PRI 67562 (Fig. 23D, F), the primary pattern is weakly visible beneath the secondary pattern. The two patterns differ in the color of emitted light. Sutural ramp with radial blotches that sometimes appear to be extensions of the primary pattern over the shoulder.

### Remarks

Maury’s [40] type specimen of *C*. *karlschmidti* is missing from the collections of the PRI (L. Skibinski, pers. comm.), so could not be compared with the shells considered here. The general shape of Maury’s figured specimen ([40], pl. 7, Fig. 14) and her description, however, closely match the shells tentatively referred here to *C*. *karlschmidti*. The coloration pattern of *C*. *karlschmidti* is very similar to that of *C*. *garrisoni* sp. nov. (see below). *Conus karlschmidti* differs from *C*. *garrisoni*, however, in having far fewer tuberculate spire whorls and having a narrower last whorl. Among extant western Atlantic species, *C*. *karlschmidti* is similar in shell shape and coloration pattern to some specimens of *C*. *amphiurgus* Dall, 1889 (e.g., [8], pl. 60, Figs. 6–8), though seems to lack the spiral rows of dots present in some specimens of the modern species. Puillandre et al. [2] assigned *C*. *amphiurgus* to the subgenus *Dauciconus*, a designation that is followed here for *C*. *karlschmidti*.

***Conus* (*Dauciconus*) *garrisoni* Hendricks sp. nov**.

urn:lsid:zoobank.org:act:17820854-5146-45DA-8035-844D1EFC4DAA

Fig. 24, S2 Table

**Fig 24:**
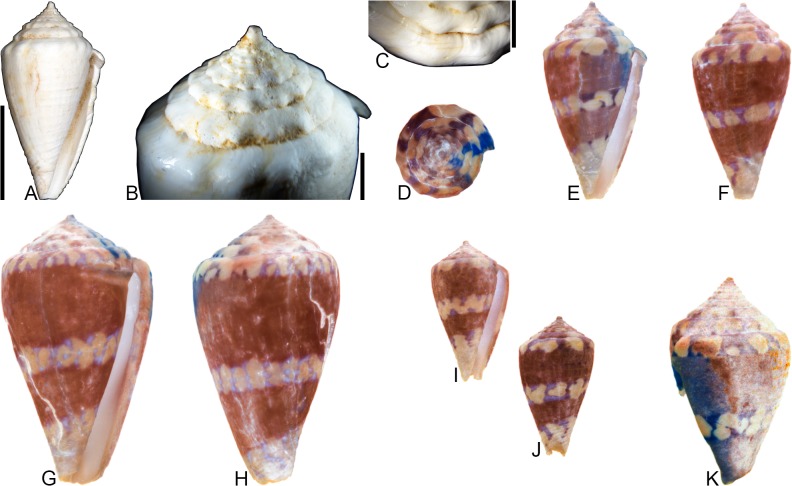
*Conus* (*Dauciconus*) *garrisoni* Hendricks sp. nov. All specimens are from locality station TU 1422 (Cercado Fm.). (A-F) PRI 66166 (holotype), SL 19.8 mm; (G-H) PRI 67197 (paratype), SL 27.2 mm; (I-J) PRI 67194 (paratype), SL 14.2 mm; (K) PRI 67196 (paratype), SL 20.8 mm. (D-K) are reversed images of specimens photographed under UV light. Scale bar to the left of (A) is 1 cm and pertains to (A, D-K); (B, C) scale bars are 2 mm.

### Material examined

**Holotype**: PRI 66166. **Paratypes**: PRI 67194, PRI 67196–67197. All type specimens are from TU station 1422.

### Type locality and horizon

TU station 1422: Arroyo Bellaco, Dominican Republic; upper Miocene Cercado Formation.

### Description

#### Shell size

Shell moderately small (largest observed specimen, PRI 67197, is 27.2 mm).

#### Last whorl

Conical (RD 0.66–0.67, μ = 0.66; PMD 0.87–0.92, μ = 0.89; n = 3); outline convex, except at anterior third, which is slightly concave. Shoulder angulate to subangulate, nearly smooth in mature specimens. Widest part of shell at or just below shoulder. Aperture uniform in width from base to shoulder. Siphonal notch absent. Fine spiral threads extending from base to shoulder in smaller specimens, restricted to anterior quarter in larger specimens.

#### Spire whorls

Spire height moderate (RSH 0.15–0.22; n = 3); outline straight to slightly sigmoidal. Protoconch multispiral, diameter 0.8 mm (based on PRI 66166). Tubercles present on first 6 postnuclear whorls, diminishing thereafter. Sutural ramp slightly sigmoidal in early whorls, convex in later whorls, ornamentation absent. Subsutural flexure symmetrical, depth about equal to width.

#### Coloration pattern

Two noninteracting patterns present. The primary (base) pattern consists of irregularly shaped and sometimes branching axial blotches that span the last whorl. The secondary pattern consists of two broad spiral bands that cover a majority of the last whorl, but not the center region. The two patterns differ in the color of emitted light. Axial markings associated with the primary pattern extend onto the sutural ramp.

### Etymology

Named in honor of American abolitionist and suffragist William Lloyd Garrison (1805–1879).

### Remarks

*Conus garrisoni* is not similar to co-occurring fossil species, but is very similar in shell morphology and coloration pattern to the extant, and highly variable (see [8]), western Atlantic species *Conus cardinalis* Hwass in Bruguière, 1792. An important difference appears to be the protoconch, which is multispiral in *C*. *garrisoni*, but paucispiral in *C*. *cardinalis* (Kohn [8] reported *C*. *cardinalis* to have 1.25–2 protoconch whorls). Tucker and Tenorio (2009) assigned *C*. *cardinalis* to the genus *Purpuriconus* da Motta, 1991, which Puillandre et al. [2] recognized as synonymous with the subgenus *Dauciconus*. While the phylogenetic context of *C*. *cardinalis* has not yet been formally explored, the strong similarity between *C*. *garrisoni* and *C*. *cardinalis* supports the assignment of the fossil species to this subgenus.

***Conus* (*Dauciconus*?) *zambaensis* Hendricks sp. nov**.

urn:lsid:zoobank.org:act:72CF869C-1094-4C5A-A5D6-270A923DA706

Fig. 25, S2 Table

**Fig 25:**
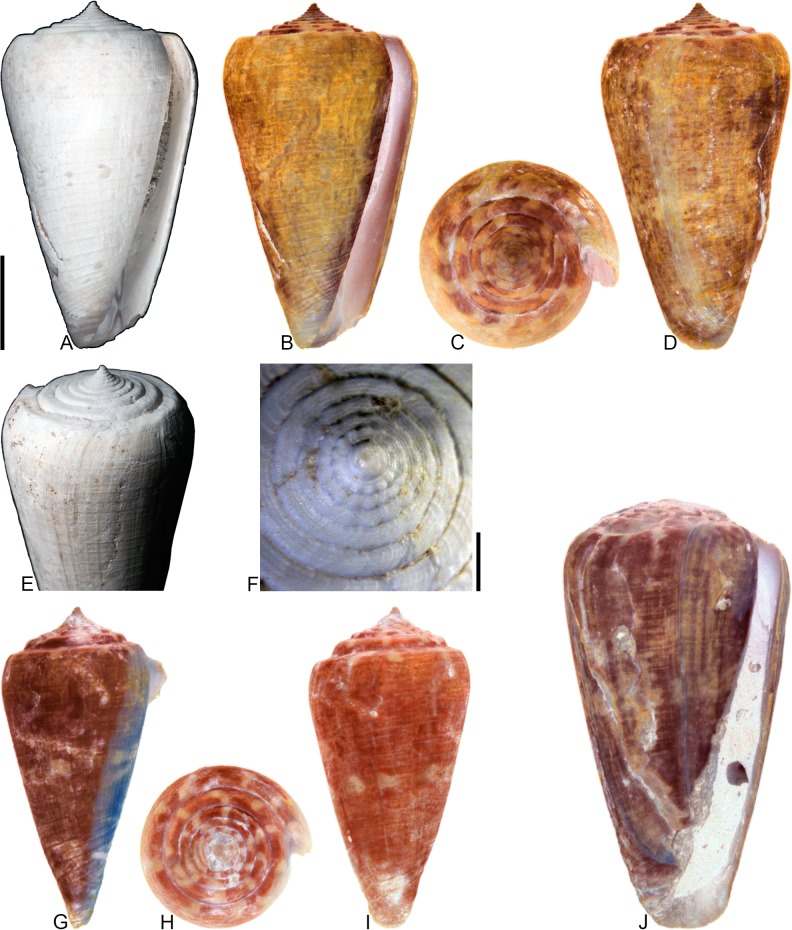
*Conus* (*Dauciconus*?) *zambaensis* Hendricks sp. nov. All specimens are from locality station TU 1354 (Gurabo Fm.). (A-F) PRI 67488 (holotype), SL 34.8 mm; (G-I) PRI 67491 (paratype), SL 32.5 mm; (J) PRI 66173 (paratype), SL 43.1 mm. (B-D, G-J) are reversed images of specimens photographed under UV light. Scale bar to the left of (A) is 1 cm and pertains to images (A-E, G-J); (F) scale bar is 1 mm.

### Material examined

**Holotype**: PRI 67488. **Paratypes**: PRI 66173, PRI 67489–67501. All type specimens are from TU station 1354.

### Type locality and horizon

TU 1354: Cañada de Zamba, Dominican Republic; lower Pliocene Gurabo Formation.

### Description

#### Shell size

Shell medium sized (largest observed specimen, PRI 66173, is 43.2 mm).

#### Last whorl

Typically conical, but sometimes ventricosely conical (RD 0.56–0.61; μ = 0.59; PMD 0.84–0.88, μ = 0.86; n = 7); outline convex, except at anterior quarter, which may be slightly concave. Shoulder angulate, smooth. Widest part of shell below shoulder. Aperture slightly wider at base than near shoulder. Siphonal notch absent. Spiral threads on anterior half, diminishing towards shoulder.

#### Spire whorls

Spire height low (RSH 0.06–0.09; μ = 0.08); outline typically concave to sigmoidal, sometimes convex; whorls may be stepped. Protoconch diameter ca. 0.7 mm (based on PRI 67488), number of whorls unknown. First 2 postnuclear whorls tuberculate, diminishing thereafter. Sutural ramp flat to slightly sigmoidal, several spiral threads present on all but the last couple of whorls in mature specimens. Subsutural flexure nearly symmetrical, depth about 1.2x width.

#### Coloration pattern

Two weakly interacting patterns present. The primary (base) pattern consists of a nearly solid ground pattern, with the exception of thin band near the middle of the last whorl where this pigmentation is discontinuous, resulting in a band of unpigmented, irregularly shaped blotches. The secondary pattern—visible only on a portion of the ventral surface of PRI 67491 (Fig. 25G)—consists of at least 12 spiral rows of dots; spaces between these are sometimes unpigmented, including when they intersect the primary pattern (these unpigmented spaces are the evidence that a weak interaction between the two patterns is present). The two patterns differ in the color of emitted light. Sutural ramp with blotches that sometimes correspond with the shape of the subsutural flexure.

### Etymology

Named for the type locality, Cañada de Zamba, Dominican Republic.

### Remarks

*Conus zambaensis* is somewhat similar in shell morphology to *Conus franklinae* sp. nov.; for differences separating the two species, see remarks for the latter taxon. The shell shape of *C*. *zambaensis*, as well as the presence of spiral threads covering much of the last whorl, are also reminiscent of the extinct species *Conus miamiensis* Petuch, 1986, which is known from the Plio-Pleistocene of Florida. *Conus miamiensis*, however, has a different coloration pattern (see [11]). Among extant taxa, *C*. *daucus* has a shape and coloration pattern that resembles *C*. *zambaensis*. All of the morphometric values reported here for *C*. *zambaensis* (RD, PMD, and RSH) are within the ranges given for *C*. *daucus* by Kohn [8]. Furthermore, *C*. *daucus*—as circumscribed by Kohn [8]—also exhibits a solid base color that is interrupted by an unpigmented, narrow band in the middle of the last whorl, as well as spiral rows of dots. One difference between *C*. *zambaensis* and *C*. *daucus* is that the fossil species has spire whorls that are often stepped, while this is not typical in *C*. *daucus* (see [8]). Puillandre et al. [2] assigned *C*. *daucus* to the subgenus *Dauciconus*, a designation that is tentatively followed here for *C*. *zambaensis*.

#### Subgenus *Spuriconus* Petuch, 2003

Type species: *Conus spurius* Gmelin, 1791.

***Conus* (*Spuriconus*) *spurius* Gmelin, 1791** [53]

Fig. 26A-F,K

**Fig 26:**
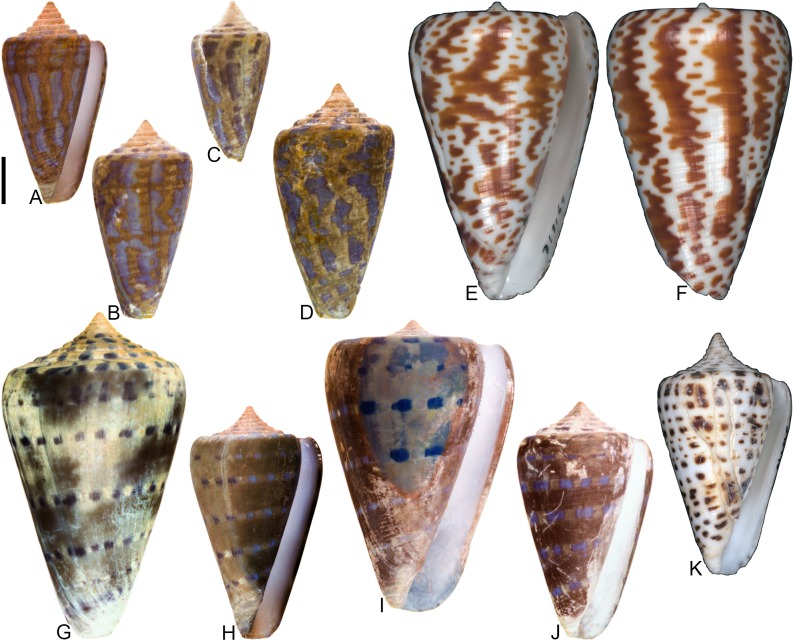
*Conus* (*Spuriconus*) *spurius* Gmelin, 1791 (A-F, K) and *Conus* (*Spuriconus*) *humerosus* Pilsbry, 1921 (G-J). Fossil specimens are from locality stations TU 1422 (Cercado Fm.), TU 1215 (Gurabo Fm.), and TU 1354 (Gurabo Fm.); modern *C*. *spurius* are from Saint Croix Island, United States Virgin Islands (E-F) and offshore of Columbia, near the Venezuelan border (K). (A) PRI 66178, TU 1354, SL 40.6 mm; (B) PRI 67542, TU 1354, SL 40.2 mm; (C) PRI 67543, TU 1354, SL 32.4 mm; (D) PRI 67544, TU 1354, SL 47.1 mm; (E-F) ANSP 313169, SL 63.3 mm (estimated from digital image); (G) PRI 66140, TU 1215, SL 65.3 mm; (H) PRI 67550, TU 1215, SL 46.9 mm; (I) PRI 66154, TU 1422, SL 59.1 mm; (J) PRI 66177, TU 1354, SL 47.9 mm; (K) ANSP 398448, SL 51.5 mm (estimated from digital image). Scale bar is 1 cm and pertains to all images.

*Conus spurius* Gmelin, 1791 [53], p. 3396; Hendricks, 2009 [11], p. 19–21, pl. 3, Figs. 1–14, pl. 4, Figs. 1–14; Kohn, 2014 [8], p. 345–357, pl. 97, Figs. 1–12, pl. 98, Figs. 1–16, pl. 99, Figs. 1–16, pl. 100, Figs. 1–17.

*Conus* (*Lindaconus*) *spurius* Gmelin, Puillandre et al., 2015 [2], p. 10.

*Spuriconus spurius* (Gmelin), Petuch, 2003 [54], p. 294; Tucker and Tenorio, 2009 [34], p. 121–122.

### Material examined

PRI 66178, PRI 67542–67544 (TU station 1354).

### Coloration pattern

One pattern present. Pattern consists of at least 4 spiral rows of heavily pigmented blotches that often coalesce to form axial streaks; these are usually visible in regular light. Sutural ramp with blotches. (Note: this color pattern description applies only to the Dominican fossil *C*. *spurius* studied here.)

### Remarks

In the Dominican Neogene, *Conus spurius*, an extant species [8], can only be confused with *C*. *humerosus* Pilsbry, 1921 (Figs. 3, 26G-J). *Conus humerosus* was originally described by Pilsbry [39] as a subspecies of *C*. *proteus*, which is a name synonymous with *C*. *spurius* (see [8]). *Conus humerosus* is tentatively treated here as a species distinct from *C*. *spurius*; while the two species are consistent in most discrete aspects of shell morphology, their coloration patterns consistently differ, including in sympatry (both species occur at TU station 1354). In particular, *C*. *humerosus* has 5–7 spiral rows of square-shaped spots separated by regions lacking pigmentation. Furthermore, *C*. *humerosus* may have two patterns of shell pigmentation, whereas *C*. *spurius* only has one.

As recently documented by Kohn [8], modern *C*. *spurius* show a great deal of variation in their pigmentation pattern. The bold, spirally-arranged axial streaks documented here in Dominican fossil *C*. *spurius* are not known from the modern fauna, though modern individuals do sometimes show blotches that have coalesced to form axial streaks (e.g., Fig. 26E,F). On the other hand, some modern specimens also show spiral rows of square-shaped spots (e.g., Fig. 26K) that are more similar to the pattern documented here for *C*. *humerosus* than for *C*. *spurius* (importantly, though, *C*. *humerosus* has fewer such rows of spots).

*Conus spurius* has a substantial fossil record in tropical America [42] and the southeastern United States [11]. Kohn [8] commented on the fossil record of *C*. *spurius* and noted that the species may “hold the distinction as the Recent species with the longest fossil record in the genus” (p. 356). The presence of *C*. *spurius* in the lower Pliocene Gurabo Fm. (TU station 1354) sets a minimum age for the species at ca. 4.9 Ma, though it may extend back to the Miocene based on its reported occurrences in Venezuela [55] and Panama [42].

This taxon was included in the phylogenetic analysis of Puillandre et al. [1] and was assigned to the subgenus *Lindaconus* Petuch, 2002 [2], the type species of which is *Conus lindae* Petuch, 1987 [2]. This subgeneric assignment is problematic, however, because the recent systematic revision of Kohn [8] recognized *C*. *lindae* as a synonym of *C*. *flavescens* Sowerby I, 1834, a species that Puillandre et al. [2] assigned to the subgenus *Dauciconus*. Tucker and Tenorio [34] assigned *C*. *spurius* to the genus *Spuriconus* Petuch, 2003 and this assignment is followed here, albeit at the subgeneric level.

***Conus* (*Spuriconus*) *humerosus* Pilsbry, 1921** [39]

Fig. 26G-J

*Conus proteus humerosus* Pilsbry, 1921 [39], p. 332, pl. 21, Fig. 4.

### Material examined

**Holotype**: ANSP 2548. **Other specimens**: PRI 66154 (TU station 1422); PRI 66140, PRI 67550 (TU station 1215); PRI 66177 (TU station 1354).

### Coloration pattern

Two weakly interacting patterns possibly present. The primary (base) pattern might consist of a solid, undifferentiated ground pattern that extends over the entire shell. The secondary pattern consists of 5–7 spiral rows of heavily pigmented, square-shaped spots; these are usually visible in regular light. Interaction between the two patterns occurs when the spiral spots intersect the base pattern, resulting in unpigmented spaces between the spiral dots (these unpigmented spaces are the evidence that a solid ground pattern may be present). The two patterns differ in the color of emitted light. Sutural ramp with blotches.

### Remarks

Among Dominican cone snail fossils, *Conus humerosus* can only be confused with *C*. *spurius* Gmelin, 1791; see remarks associated with *C*. *spurius* for a comparison between the two forms. Additional work will be required to determine whether *C*. *humerosus* is truly a distinct species from *C*. *spurius* (as suggested here), or whether it is a variation of *C*. *spurius* (as suggested first by Pilsbry [39], who described it as a subspecies of *C*. *proteus*, a synonym of *C*. *spurius*). Given its close association with *C*. *spurius*, *C*. *humerosus* is also assigned here to the subgenus *Spuriconus*.

***Conus* (*Spuriconus*?) *kaesleri* Hendricks sp. nov**.

urn:lsid:zoobank.org:act:11665A29-6D03-440E-82FE-E3515B7D7808

Fig. 27, S2 Table

**Fig 27:**
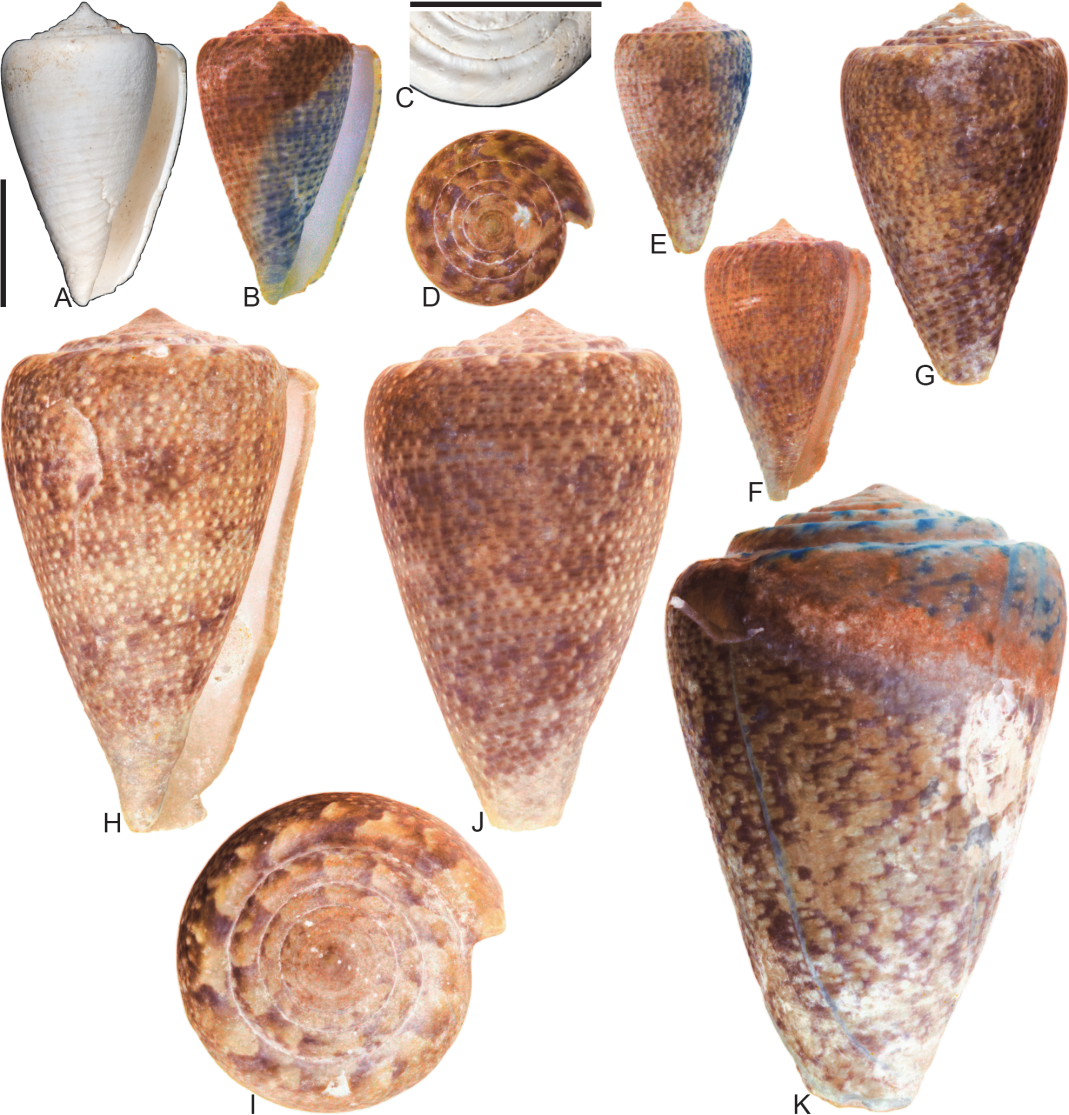
*Conus* (*Spuriconus*?) *kaesleri* Hendricks sp. nov. All specimens are from locality station TU 1354 (Gurabo Fm.). (A-D) PRI 66185 (holotype), SL 22.6 mm; (E) PRI 67668 (paratype), SL 18.8 mm; (F) PRI 67669 (paratype), SL 21.2 mm; (G) PRI 67667 (paratype), SL 28.5 mm; (H-J) PRI 67666 (paratype), SL 38.7 mm; (K) PRI 67670 (paratype), SL 46.5 mm. (B, D-K) are reversed images of specimens photographed under UV light. Scale bar to the left of (A) is 1 cm and pertains to (A-B, D-K); (C) scale bar is 1 cm.

### Material examined

**Holotype**: PRI 66185. **Paratypes**: PRI 67666–67671. All type specimens are from TU station 1354.

### Type locality and horizon

TU 1354: Cañada de Zamba, Dominican Republic; lower Pliocene Gurabo Formation.

### Description

#### Shell size

Shell medium sized (largest observed specimen, PRI 67670, is 46.5 mm).

#### Last whorl

Typically conical or broadly conical, but sometimes ventricosely conical (RD 0.66–0.72, μ = 0.69; PMD 0.83–0.89, μ = 0.87; n = 5); outline convex, except at anterior quarter, which may be slightly concave. Shoulder subangulate, smooth. Widest part of shell below shoulder. Aperture uniform in width from base to shoulder. Siphonal notch absent. Spiral threads, which are often beaded, on anterior half, diminishing towards shoulder.

#### Spire whorls

Spire height low to moderate (RSH 0.09–0.14; μ = 0.11, n = 5); outline concave to slightly sigmoidal. Protoconch unknown. Early postnuclear whorls unknown; tubercles absent from preserved whorls. Sutural ramp sigmoidal, unornamented. Subsutural flexure asymmetrical, depth about 0.9x width.

#### Coloration pattern

In most specimens, two noninteracting patterns present. The primary (base) pattern consists of 2–4 discontinuous spiral bands. The secondary pattern consists of about 20–40 spiral rows of dots or dashes; spaces between these are sometimes unpigmented. The two patterns differ slightly in the color of emitted light. Sutural ramp with radial blotches that sometimes correspond with the shape of the subsutural flexure. One eroded specimen—PRI 67670—shows a different coloration pattern: its last whorl is covered in a network of small, chevron-shaped markings (Fig. 27K).

### Etymology

Named in honor of University of Kansas paleontologist Roger L. Kaesler (1937–2007).

### Remarks

Among other Neogene cone snail fossils from the Dominican Republic, *C*. *kaesleri* is similar to *C*. *lombardii* sp. nov. (see below) and *C*. *spurius* Gmelin, 1791 (see above), which is an extant species. All three species have somewhat similar shell shapes and lack ornamentation on their sutural ramps. The coloration pattern of *C*. *spurius*, however, is very different from either of the newly described species, both of which have numerous spiral rows of dots and dashes on their last whorls. *Conus kaesleri* (which is only known from the Gurabo Fm.) and *C*. *lombardii* (which is only known from the Cercado Fm.) are not known to occur together, but are likely closely related. Several features separate the two species, however. First, the width of the aperture is uniform from the base to the shoulder in *C*. *kaesleri*, but is slightly wider near the base in *C*. *lombardii*. Second, specimens of *C*. *kaesleri* tend to have lower spires (average RSH is 0.11) than specimens of *C*. *lombardii* (average RSH is 0.17). Finally, the coloration patterns vary slightly, as the discontinuous spiral bands present in *C*. *kaesleri* are absent in *C*. *lombardii*. Interestingly, the same networked, chevron-like pattern described above for the eroded specimen PRI 67670 is also present on an eroded specimen of *C*. *lombardii* (PRI 67213). This suggests that both species may have had an additional level of coloration patterning that was not expressed at the surface of the last whorl of the shell.

Puillandre et al. [2] assigned *C*. *spurius* to the subgenus *Lindaconus*, but it is here assigned to the subgenus *Spuriconus* (see remarks for *C*. *spurius*). Given their strong similarities to *C*. *spurius*, *C*. *kaesleri* and *C*. *lombardii* are tentatively also assigned to *Spuriconus*.

***Conus* (*Spuriconus*?) *lombardii* Hendricks sp. nov**.

urn:lsid:zoobank.org:act:8BD8086D-09BB-4D38-BD3D-DCADEEDCC9F6

Fig. 28, S2 Table

**Fig 28:**
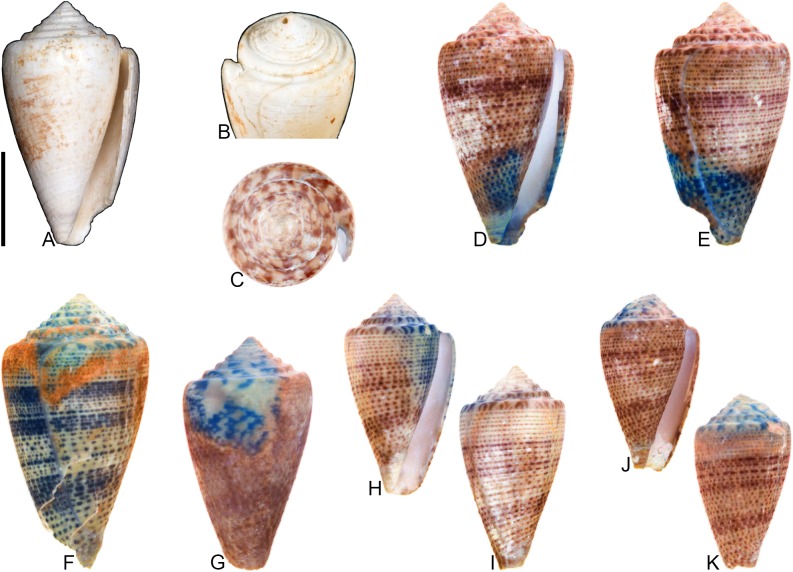
*Conus* (*Spuriconus*?) *lombardii* Hendricks sp. nov. All specimens are from locality station TU 1422 (Cercado Fm.). (A-E) PRI 67207 (holotype), SL 24.7 mm; (F) PRI 66165 (paratype), SL 27.4 mm; (G) PRI 67213 (paratype), SL 23.7 mm; (H-I) PRI 67218 (paratype), SL 20.5 mm; (J-K) PRI 67217 (paratype), SL 17.9 mm. (C-K) are reversed images of specimens photographed under UV light. Scale bar is 1 cm and pertains to all images.

### Material examined

**Holotype**: PRI 67207. **Paratypes**: PRI 66165, PRI 67189, 67208–67224. All type specimens are from TU station 1422.

### Type locality and horizon

TU 1422: Arroyo Bellaco, Dominican Republic; upper Miocene Cercado Formation.

### Description

#### Shell size

Shell moderately small (largest observed specimen, PRI 67215, is 29.5 mm; this specimen is slightly damaged).

#### Last whorl

Typically conical, but sometimes ventricosely conical (RD 0.65–0.70, μ = 0.67; PMD 0.83–0.90, μ = 0.88; n = 11); outline slightly convex. Shoulder angulate, smooth. Widest part of shell just below shoulder. Aperture slightly wider at base than near shoulder. Siphonal notch absent. Spiral threads, which are often beaded, on anterior half, diminishing towards shoulder.

#### Spire whorls

Spire height moderate (RSH 0.14–0.20, μ = 0.17; n = 11); outline straight to slightly concave. Protoconch unknown. One specimen (PRI 67215) shows possible evidence of one tuberculate postnuclear whorl; tubercles otherwise absent from all spire whorls. Sutural ramp sigmoidal, unornamented. Subsutural flexure asymmetrical; depth about 1.7–2x width.

#### Coloration pattern

Two noninteracting patterns present. The primary (base) pattern, partially exposed on eroded posterior dorsal surface of last whorl on PRI 67213 (Fig. 28G), consists of a network of small, chevron-shaped markings. The secondary pattern consists of 27–41 (number increases with shell size) rows of spiral dots or dashes extending from base to shoulder; the dashes are often concentrated, forming several false spiral bands. The two patterns differ in the color of emitted light. Sutural ramp with radial blotches; PRI 67213 suggests that these are extensions over the shoulder of the primary pattern on the last whorl and hence are the only features of the primary pattern that are typically visible on most shells.

### Etymology

Named in honor of Vincent Lombardi (1913–1970), former coach of the Green Bay Packers who led the team to victory in Super Bowl I and II.

### Remarks

*Conus lombardii* is similar to two other fossil species from the Neogene of the Dominican Republic: *C*. *kaesleri* sp. nov. and *C*. *spurius* Gmelin, 1791. See remarks associated with *C*. *kaesleri* for important differences that separate these three species, as well as justification for the tentative placement of *C*. *lombardii* in the subgenus *Spuriconus*.

**Subgenus *Lautoconus* Monterosato, 1923**

Type species: *Conus ventricosus* Gmelin, 1791.

***Conus* (*Lautoconus*?) *carlottae* Hendricks sp. nov**.

urn:lsid:zoobank.org:act:BD5518E2-C191–4985-A736-AC17AF0243EC

Fig. 29, S2 Table

**Fig 29:**
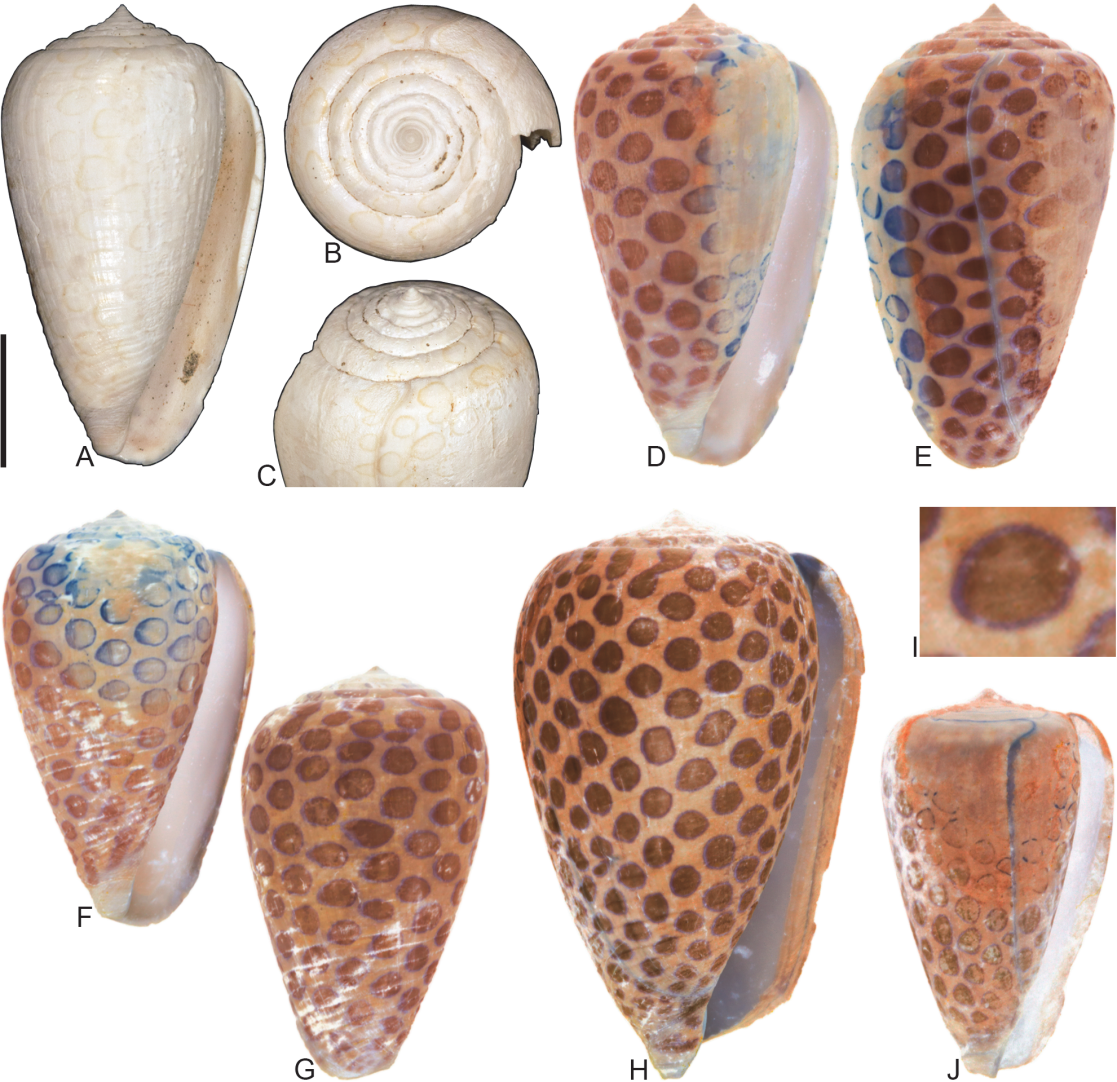
*Conus* (*Lautoconus*?) *carlottae* Hendricks sp. nov. Specimens are from locality stations TU 1422 (Cercado Fm.) and TU 1354 (Gurabo Fm.). (A-E) PRI 66199 (holotype), TU 1354, SL 33.0 mm; (F-G) PRI 67154 (paratype), TU 1354, SL 29.7 mm; (H-I) PRI 66161 (paratype), TU 1422, SL 40.2 mm; (J) PRI 66198 (paratype), TU 1422, SL 27.8 mm. (D-J) are reversed images of specimens photographed under UV light. Scale bar to left of (A) is 1 cm and pertains to images (A-H, J); note that (I) is a magnified view of one of the pigmented spots on (H).

### Material examined

**Holotype**: PRI 66199 (TU station 1354). **Paratypes**: PRI 66161 and 66198 (TU station 1422); PRI 66200–66201 and PRI 67154–67163 (TU station 1354).

### Type locality and horizon

TU 1354: Cañada de Zamba, Dominican Republic; lower Pliocene Gurabo Formation.

### Other locality and horizon

TU 1422: Arroyo Bellaco, Dominican Republic; upper Miocene Cercado Formation.

### Description

#### Shell size

Shell medium sized (largest observed specimen, PRI 66161, is 40.2 mm).

#### Last whorl

Ventricosely conical (RD 0.65–0.69, μ = 0.66; PMD 0.79–0.84, μ = 0.81; n = 10); outline convex. Shoulder rounded, smooth. Widest part of shell below shoulder. Aperture slightly wider at base than near shoulder. Siphonal notch absent; anterior-most end of lip extends beyond columella. Broad and anteriorly sloping spiral ribs on anterior half that diminish toward the shoulder.

#### Spire whorls

Spire height low to moderate (RSH = 0.10–0.18, μ = 0.12; n = 10); outline sigmoidal. Protoconch unknown. Tubercles absent from all spire whorls. Sutural ramp flat in early whorls, convex in later whorls, with several very fine spiral grooves (see PRI 66199); one specimen (PRI 67159) shows evidence of two spiral threads on the earliest postnuclear whorls. In later whorls, the intersection of the sutural ramp with the previous whorl forms a channel along the suture. Subsutural flexure asymmetrical, depth 1–1.5x width.

#### Coloration pattern

One pattern present. Pattern consists of round- to teardrop-shaped spots that are situated in a loosely hexagonal packing arrangement over the entirety of the last whorl. The spots are ringed—most prominently on their abaxial sides—by fluorescing regions that differ in emitted wavelength from the enclosed spots (see Fig. 29I). The last whorl pattern extends over the shoulder onto the sutural ramp.

### Etymology

Named for Carlotta Maury (1874–1938) in honor of her important contributions to paleontology, particularly her work on Neogene mollusks from the Dominican Republic [40]. For a biography of Maury’s life, see the recent work by Arnold [56, 57]. (Note: Finlay [58] honored Maury with the nomen novum *C*. *mauryi* to replace the occupied name *C*. *ornatus* (Röding, 1798), which Maury [40] applied to a very different species from the Dominican Republic.)

### Remarks

The distinctive shell coloration pattern exhibited by *C*. *carlottae* is unknown among modern cones snails. In overall shell form, however, *C*. *carlottae* bears resemblance to some of the extant species that Tucker and Tenorio [34] assigned to the genus *Varioconus* da Motta, 1991 and that Puillandre et al. [2] attributed to the subgenus *Lautoconus* Monterosato, 1923; today these species occur along the west coast of Africa. The extinct species *Conus yaquensis* Gabb, 1873 (e.g., http://neogeneatlas.org/species/conus-yaquensis-2/), known from the upper Pliocene Tamiami Fm. of Florida [11], is somewhat similar in shell morphology, but has the opposite coloration pattern: a net-like reticulated pattern that forms unpigmented dots. The pattern of *C*. *yaquensis* is similar to that of some specimens of *C*. *mercator* Linnaeus, 1758 (e.g., http://www.coneshell.net/Pages/c_mercator.htm), an extant species assigned by Puillandre et al. [2] to the subgenus *Lautoconus*. This subgeneric assignment is tentatively followed here for *C*. *carlottae*.

### Additional Forms

**Fig 30:**
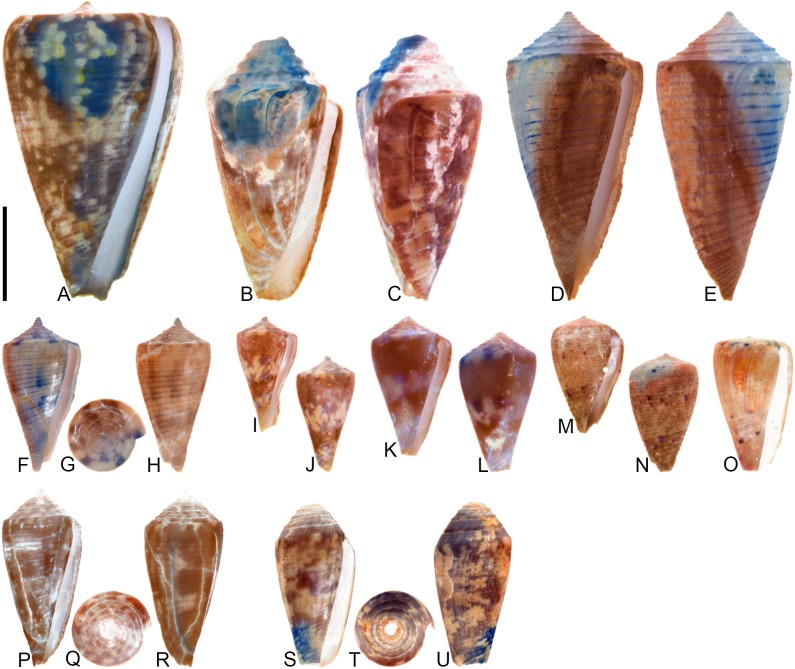
Unidentified Conidae spp. Specimens are from locality stations TU 1422 (Cercado Fm.), TU 1215 (Gurabo Fm.), and TU 1354 (Gurabo Fm.). (A) Conidae sp. 1, PRI 66169, TU 1422, SL 30.5 mm; (B-C) Conidae sp. 2, PRI 66186, TU 1354, SL 26.6 mm; (D-E) Conidae sp. 3, PRI 67243, TU 1354, SL 29.2 mm; (F-H) Conidae sp. 4, PRI 67566, TU 1215, SL 16.3 mm; (I-J) Conidae sp. 5, PRI 67234, TU 1422, SL 11.8 mm; (K-L) Conidae sp. 6, PRI 67233, TU 1422, SL 14.1 mm; (M-N) Conidae sp. 7, PRI 66167, TU 1422, SL 11.9 mm; (O) Conidae sp. 8, PRI 67571, TU 1215, SL 14.3 mm; (P-R) Conidae sp. 9, PRI 66149, TU 1215, SL 18.8 mm; (S-U) Conidae sp. 10, PRI 66145, TU 1215, SL 17.4 mm. All are reversed images of specimens photographed under UV light. Scale bar is 1 cm and pertains to all images.

**Fig 31:**
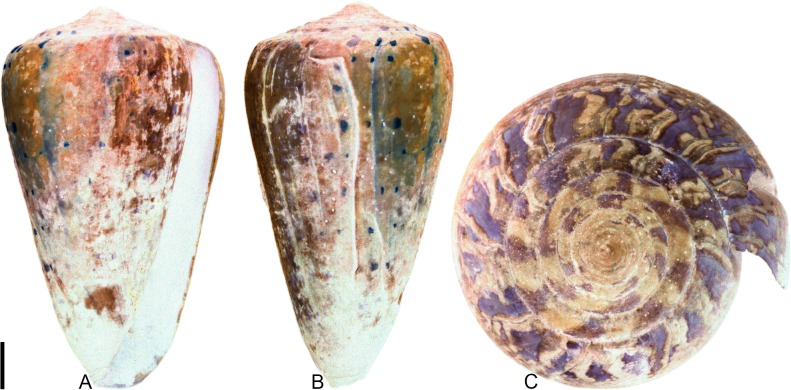
Unidentified Conidae spp. Specimens are from locality station TU 1354 (Gurabo Fm.). (A-B) Conidae sp. 11, PRI 67253, SL 76.4 mm; (C) Conidae sp. 12, PRI 66172, MD 68.2 mm (only spire is preserved). All are reversed images of specimens photographed under UV light. Scale bar is 1 cm and pertains to all images.

While most of the specimens from TU stations 1422, 1215, and 1354 could be assigned to either previously described species or definitively recognized as the new species described herein, 34 specimens remain taxonomically unassigned. In some cases, the poor preservational states of the specimens prevent them from being further studied. Many of the specimens, however, are well preserved and may represent additional undescribed diversity. They were not described here, however, because they are either currently represented by fewer than three known specimens (the threshold used in this study; see above) or could potentially be juvenile forms of taxa that have already been described (Kohn [8] also encountered this problem and discussed it on p. 382). Nevertheless, those that have well preserved coloration patterns are figured here (Figs. 30, 31), but are not further described or remarked upon at this time.

## Discussion

The three coral reef-associated fossil assemblages considered here (TU stations 1422, 1215, and 1354) collectively include a minimum of 28 cone snail species, 13 of which had not been previously described (Table 1). To put this number in perspective, Kohn’s [8] recent systematic revision of extant cone snails from the southeastern United States and Caribbean recognized a total of 53 valid species (from a pool of 263 available names) and Hendricks [11] recognized 23 total fossil species from the Plio-Pleistocene of the southeastern United States. It is not yet known how many total species of cone snails occur in the Neogene fossil record of the Dominican Republic, but many previously described species were not observed in the three assemblages studied here, suggesting that the total diversity of the fauna—which spans the late Miocene to early Pliocene (6.6-3.5 Ma; McNeill et al., 2012)—may significantly exceed the 28 species documented here (nearly 50 species names have been applied to the fauna in the published literature).

Alan Kohn and his colleagues have extensively studied the community ecology of cone snails on modern Indo-Pacific coral reefs (e.g., [59–62]). This study allows comparisons to be made between cone snail diversity patterns on modern and fossil coral reef systems for the first time. Kohn [60] showed that modern Indo-Pacific reefs support an average of 13.7 cone snail species (range 9–24 species), though a later work [62] documented 36 co-occurring species at a reef off the coast of Papua New Guinea. The minimum number of species at TU stations 1422, 1215, and 1354 ranged from 14–16 (Table 1), values that are remarkably consistent with Kohn [60]. It is worth noting, however, that the fossil reef localities each represent a much greater amount of time (e.g., ca. 0.6 My in the case of TU station 1215), so the number of species that may have been co-existing on each reef during any given period of time may have been less.

All of the 28 species documented from TU stations 1422, 1215, and 1354 include at least one specimen that exhibits a preserved coloration pattern when illuminated by UV light. The great diversity of these revealed coloration patterns—especially the similarities they share with extant species—allow most to be assigned to particular cone snail clades [1], pending their inclusion in an explicit phylogenetic analysis. In total, the 28 species were assigned (or tentatively assigned) to three major clades of cone snails and many subclades: *Profundiconus* (1 species), *Conasprella* (5 species assigned to at least 2 subgenera), and *Conus* (22 species assigned to at least 7 subgenera). The Dominican Neogene cone snail fauna can therefore be considered to be phylogenetically diverse, which has also been demonstrated for the extant western Atlantic fauna [1, 64].

Presuming that these phylogenetic assignments are correct, they provide useful information about the minimum ages of the clades to which these fossil species may belong and thus have the potential to serve as useful calibration points in future molecular clock-based estimates of the divergence times of different cone snail lineages. As just one example, Duda and Kohn [63] estimated that *Conasprella tornata* diverged from the clade that includes *C*. *mahogani* and *C*. *ximenes* about 3 Ma. The newly described fossil species *Conasprella ageri* (Fig. 8A-P) was likely a close relative of *C*. *tornata* and occurs abundantly at TU station 1354, which is estimated to have an age of ca. 4.9 Ma. If correct, this extends the age of clade (*C*. *tornata*(*C*. *mahogani*,*C*. *ximenes*)) back to at least 4.9 Ma, and perhaps back to at least 6.0 Ma if the two shells from TU station 1422 that are questionably assigned to *C*. *ageri* are indeed that species. Table 2 summarizes the implied minimum ages of 17 extant species lineages and clades based on the fossil records of DR Neogene cone snails that are hypothesized to be close relatives (see remarks associated with individual fossil species for justification).

**Table 2:** Minimum ages of clades implied by the fossil record of Dominican Neogene cone snail fossils.

Dominican Fossil Species	Similar Extant Species	Minimum Age of Clade
*Conasprella* (*Dalliconus*) cf. *sauros* (Garcia, 2006)	*C*. sauros*	4.8 Ma (TU 1215)
*Conasprella* (*Ximeniconus*) *ageri* sp. nov.	(*C*. *tornata*(*C*. *mahogani*,*C*. *ximenes*))	4.9 Ma (TU 1354)
*Conasprella* (*Ximeniconus*) *cercadensis* (Maury, 1917) and *C*. (*X*.) *kitteredgei* (Maury, 1917)	(*C*. *perplexa*,*C*. *puncticulata*)	6.0 Ma (TU 1422)
*Conasprella stenostoma* (Sowerby I, 1850)	*C*. *delessertii*	4.8 Ma (TU 1215)
*Conus* ("*Pyruconus*") *haytensis* Sowerby I, 1850	*C*. *fergusoni*	4.8 Ma (TU 1215)
*Conus* (*Atlanticonus*?) *olssoni* Maury, 1917 and *C*. (*A*.?) *franklinae* sp. nov.	*C*. granulatus*	6.0 Ma (TU 1422)
*Conus* (*Dauciconus*?) *zambaensis* sp. nov.	(*C*. *richardbinghami*(*C*. *daucus*,*C*. *jacarusoi*))	4.9 Ma (TU 1354)
*Conus* (*Dauciconus*) *furvoides* Gabb, 1873	(*C*. *vergatus*,*C*. *recurvus*)	4.9 Ma (TU 1354)
*Conus* (*Dauciconus*) *garrisoni* sp. nov.	*C*. cardinalis*	6.0 Ma (TU 1422)
*Conus* (*Dauciconus*) *karlschmidti*? Maury, 1917	(*C*. *amphiurgus*,*C*. *villepinii*)	4.8 Ma (TU 1215)
*Conus* (*Dauciconus*) *multiliratus* Böse, 1906 and *C*. (*D*.) *planiliratus* Sowerby I, 1850	*C*. *cancellatus*	4.8 Ma (TU 1215)
*Conus* (*Ductoconus*) *cashi* sp. nov.	*C*. *princeps*	6.0 Ma (TU 1422)
*Conus* (*Lautoconus*?) *carlottae* sp. nov.	*C*. *mercator*	6.0 Ma (TU 1422)
*Conus* (*Pyruconus*) *recognitus* Guppy, 1866	*C*. *patricius*	4.9 Ma (TU 1354)
*Conus* (*Spuriconus*) *spurius* Gmelin, 1791, *C*. (*S*.) *humerosus* Pilsbry, 1921, *C*. (*S*.?) *kaesleri* sp. nov., and *C*. (*S*.?) *lombardii* sp. nov.	*C*. *spurius*	6.0 Ma (TU 1422)
*Conus* (*Stephanoconus*) *gouldi* sp. nov.	(*C*. *regius*(*C*. *bartschi*,*C*. *brunneus*))	6.0 Ma (TU 1422)
*Conus* (*Stephanoconus*) *sewalli* Maury, 1917 and *C*. (*S*.) *bellacoensis* sp. nov.	(*C*. *archon*(*C*. *cedonulli*(*C*. *curassaviensis*,*C*. *mappa*)))	6.0 Ma (TU 1422)
*Conus anningae* sp. nov.	n/a	n/a
*Conus lyelli* sp. nov.	n/a	n/a
*Conus symmetricus* Sowerby I, 1850	n/a	n/a
*Profundiconus*? *hennigi* sp. nov.	n/a	n/a

See remarks associated with individual fossil species for similarities shared with extant taxa. Parenthetical notation reflects the hypothesis of species relationships published by Puillandre et al. [1]; extant species marked by an asterisk were not included in that study. Clade ages based on minimum ages of the TU locality stations studied here; for listed groups containing more than one fossil species, the oldest taxon was used to set the date (see Table 1).

Finally, several of the fossil species provide some interesting biogeographical implications for understanding the regional development of the modern western Atlantic cone snail fauna. Three extant eastern Pacific species, *Conasprella tornata*, *Conus patricius*, and *C*. *princeps* appear much more closely related to three extinct Dominican species than they do to extant western Atlantic species; respectively, these fossil species are *Conasprella ageri*, *Conus recognitus*, and *Conus cashi*. The three extant eastern Pacific species therefore provide clear examples of what Woodring [48] characterized as paciphiles: groups that were once widespread in tropical America, but that now persist only in the eastern Pacific (it should be noted that Woodring [48] himself characterized *Conus patricius* as a paciphilic species). Further study of the fossil records of these three Dominican species may help to resolve the timing of their extinction relative to the final closure of the Central American Seaway about 3 Ma [64], which may have played a role in their demise.

In the introduction to his recent monograph on western Atlantic cone snails, Alan Kohn noted that “the geologic record of the genus [*Conus*] still remains too poorly known to provide useful insights into the evolution of the region’s modern *Conus* fauna” ([8], p. 1). This statement is, unfortunately, quite true. Our understanding of the phylogenetic contexts of most of the fossil species from this region relative to the modern fauna is nearly non-existent, yet is key to attaining a comprehensive understanding of the evolution of this remarkable clade of gastropods. One way of achieving this goal is through “total evidence” phylogenetic analyses that combine morphological data from extant and fossil species with molecular sequence data from extant species (e.g., [65, 66]). The revealed coloration patterns documented here provide an additional character suite that will likely be useful for placing fossil cone snails into phylogenies (also see [66]). This approach—combined with the exceptional Neogene fossil record of the Dominican Republic, as well as other regional fossil deposits—has the potential to provide a detailed understanding of the evolutionary and biogeographic development of the modern western Atlantic cone snail fauna, and more generally illuminate the history of a diverse marine clade and its members’ responses to environmental changes over millions of years.

## Supporting Information

S1 Table Specimens observed. List of PRI specimens observed from locality stations TU 1422, 1215, and 1354.
(XLSX)

S2 Table Measurement data for newly described species. SL, shell length; MD, maximum diameter; AH, aperture height; HMD, height of maximum diameter; RD, relative diameter; PMD, position of maximum diameter; and RSH, relative spire height. Asterisk (*) indicates measurement values that are affected by slightly damaged shells.
(XLSX)
